# DEAD-box helicase gcDDX56 disrupts IRF3 nuclear import complex and promotes nuclear IRF3 degradation for enhancing GCRV replication

**DOI:** 10.1128/jvi.01654-25

**Published:** 2025-11-11

**Authors:** Li Li Xiao, Yang Chen, Jie Zhang, Jun Xiao, Hao Feng, Ming Xian Chang

**Affiliations:** 1 College of Life Science, Hunan Normal University 12568, Changsha, China; 2 State Key Laboratory of Breeding Biotechnology and Sustainable Aquaculture (CAS), State Key Laboratory of Freshwater Ecology and Biotechnology, Institute of Hydrobiology, Chinese Academy of Sciences 53021, Wuhan, Hubei, China; 3 College of Advanced Agricultural Sciences, University of Chinese Academy of Sciences 74519, Beijing, China; University of Kentucky College of Medicine, Lexington, Kentucky, USA

**Keywords:** grass carp DDX56, IRF3, KPNB3, grass carp reovirus

## Abstract

DEAD-box helicases play multifaceted roles in antiviral immunity, acting as both viral sensors and regulators of interferon (IFN) signaling. However, the role of DDX56 in teleost antiviral immunity remains poorly characterized. This study investigates the role of grass carp DDX56 (gcDDX56) in regulating IFN responses during grass carp reovirus (GCRV) infection. Dual-luciferase reporter assays reveal that gcDDX56 potently suppresses GCRV-induced activation of IFN1/3 promoters and RLR signaling components (RIG-I, MDA5, MAVS, TBK1, IRF3/7). Mechanistically, gcDDX56 reduces IRF3 protein levels via autophagy–lysosome-mediated degradation, confirmed by cycloheximide (CHX) chase experiments and autophagy inhibitor rescue. Nuclear protein fractionation confirmed that gcDDX56 overexpression significantly reduced nuclear and cytoplasmic IRF3 protein levels during GCRV infection. Unlike mammalian DDX56, which directly interacts with IRF3, gcDDX56 does not bind gcIRF3. Instead, it sequesters importin β3 (gcKPNB3) in the nucleus, disrupting the gcIRF3-gcKPNB3 complex required for IRF3 nuclear import. Confocal microscopy validates nuclear co-localization of gcDDX56-gcKPNB3 and competitive binding with gcIRF3. Transcriptional profiling shows that gcDDX56 suppresses the expression of type I IFNs (*ifn1*, *ifn3*), IFN-stimulated genes (*mx1*, *mx2*), and RLR pathway components induced by gcIRF3-gcKPNB3. These findings establish a novel dual mechanism for gcDDX56: autophagy-dependent IRF3 degradation and gcKPNB3-mediated nuclear import blockade. This evolutionary divergence highlights teleost-specific adaptations in antiviral immunity, providing insights into GCRV pathogenesis and potential targets for aquaculture disease control.

**IMPORTANCE:**

DEAD-box helicases exhibit versatile cellular roles in antiviral defense, functioning both as sentinels detecting viral invaders and as regulators of immune signaling. The present study reveals that teleost fish have evolved unique strategies using a related protein called gcDDX56. Unlike previously studied DEAD-box helicases, we demonstrate for the first time that gcDDX56 hijacks an importin β's (gcKPNB3) dual functions (IRF3 transport/degradation) via domain-specific interaction, acting as a “switch” to tilt gcKPNB3 toward pro-viral degradation. This novel regulatory axis (gcDDX56-gcKPNB3-IRF3) reveals a “triple hit” mechanism (cytoplasmic degradation + nuclear clearance + import blockade) that maximizes IRF3 suppression, a strategy not reported for other viruses. Beyond advancing basic knowledge of vertebrate innate immunity (expanding DEAD-box helicase/importin β functional repertoires), these findings provide actionable targets (e.g., gcDDX56-Helicase C/gcKPNB3-KAP95 interface) for developing anti-GCRV therapies, addressing a pressing need in aquaculture to mitigate GCRV-induced losses.

## INTRODUCTION

The DEAD-box (DDX) RNA helicases belonging to superfamily 2 (SF2), the largest group of eukaryotic RNA helicases of six superfamilies, are named after a conserved amino sequence (Asp-Glu-Ala-Asp/His) (1). RNA helicases play regulatory roles in a variety of cellular processes, including ATP binding, ATP hydrolysis, nucleic acid binding, and RNA unwinding activity, covering virtually all aspects of gene expression and its regulation (2–4). Intriguingly, apart from their roles in RNA metabolism, RNA helicases also actively participate in viral infection given that viruses rely heavily on host RNA helicases to mediate RNA remodeling events that are part of their replication cycle or required for viral gene expression (5). For example, DDX3 has been identified as an essential cellular factor for the replication of different viruses, including important human viruses such as human immunodeficiency virus (HIV-1) or hepatitis C virus (HCV) (6). In the meantime, several DExH/D-box helicases, such as DDX3, DDX41, DHX9, and DDX1/DDX21/DHX36 complex, have been reported to act as viral sensors, while other helicases, including DDX60, DDX60L, and DDX23, function in the activation of innate immune response (7).

To defend against invading viruses, host cells initiate innate immune responses via a set of pattern recognition receptors (PRRs), including the Toll-like receptors (TLRs), retinoic acid-inducible gene I (RIG-I)-like receptors (RLRs), and cytosolic DNA sensors, such as cyclic GMP-AMP (cGAMP) synthase (cGAS), IFI16, and DDX41 (8, 9). The RLRs comprise three core members: RIG-I (also known as DDX58), melanoma differentiation-associated gene 5 (MDA5, or IFIH1), and laboratory of genetics and physiology 2 (LGP2). These receptors primarily recognize viral double-stranded RNA or single-stranded RNA in most cells through their C-terminal RNA helicase domains (10). RIG-I and MDA5 interact with mitochondrial antiviral-signaling protein (MAVS) through CARD domains (11). Then MAVS recruits and activates TANK-binding kinase-1 (TBK1) and Inhibitor-κB kinase ε (IKKε). The interferon regulatory factor 3 (IRF3), a key transcription factor, is phosphorylated by activated TBK1 and IKKε. This phosphorylation triggers IRF3 dimerization, and the resulting dimers are subsequently translocated into the nucleus via the nuclear pore complex, where they drive the transcription of type I interferons (IFNs) (12, 13). Notably, LGP2, which lacks CARD domains, modulates this pathway through regulatory interactions with RIG-I/MDA5 or viral RNA, fine-tuning the amplitude and duration of the antiviral response (14, 15). The well-characterized importin alpha (KPNA) and importin beta (KPNB) nuclear import pathways play a crucial role in the innate immune response to viral infection by mediating the nuclear import of transcription factors, such as IRF3, NF-κB, and STAT1 (16). It is reported that IRF3 contains a nuclear localization signal (NLS) that is recognized and bound by importin-α receptors, such as KPNA2 and KPNA4, which assist IRF3 in translocating from the cytoplasm to the nucleus (17, 18).

In teleosts, several RNA helicases have been reported to be involved in the innate immune response or viral infection. Overexpression of DDX5 inhibited IFN production induced by spring viremia of carp virus (SVCV) and poly(I:C), and enhanced SVCV replication by targeting the autophagic degradation of TBK1 and disrupting the formation of TBK1-TRAF3 complex (19). Up to now, there are more and more research findings on DDX56 function. In mammals, DDX56 was found to play a part in cancer processes and had been identified as a potential therapeutic target in hepatocellular carcinoma (HCC) tumorigenesis (20, 21). Knockout or RNAi knockdown of mouse DDX56 led to ribosome dysfunction and cell lethality, suggesting that DDX56 participated in ribosome assembly (22). At the same time, it also plays a variety of roles in viral infections. Porcine DDX56 can inhibit Pseudorabies virus (PRV) replication through promoting cGAS-STING-induced IFN-β expression (23); however, mammalian DDX56 enhanced the replication of foot-and-mouth disease virus (FMDV) by inhibiting the phosphorylation of IRF3 (24). Whether piscine DDX56 participates in viral infection and its exact mechanism remains unclear.

In this study, we cloned and investigated DDX56 in grass carp (named as gcDDX56) and identified gcDDX56 as a positive regulator of GCRV infection. gcDDX56 can disrupt the interaction between gcIRF3 and gcKPNB3, which inhibits the nuclear translocation of gcIRF3. However, this single mechanism is insufficient to explain the full extent of immune suppression observed. Instead, gcDDX56 hijacks gcKPNB3 dual functions (IRF3 transport/degradation) via domain-specific interaction, which synergistically suppress IFN signaling and boost GCRV replication via a “triple hit” strategy (cytoplasmic IRF3 degradation + nuclear IRF3 clearance + import blockade).

## RESULTS

### Sequence, expression, and subcellular localization of gcDDX56

The NCBI conserved domain analysis revealed that the gcDDX56 protein harbored a conserved DEAD-box helicase domain (DEAD) in its N-terminal region and a Helicase_C domain in its C-terminal region. These structural features are evolutionarily conserved across mammalian species and other model organisms (Fig. S1A). To investigate evolutionary relationships, a phylogenetic tree was constructed using vertebrate DDX56 orthologs. Phylogenetic tree analysis demonstrated that gcDDX56 clustered within the Cypriniformes, sharing the highest evolutionary proximity with *Megalobrama amblycephala* (Fig. S1B). Sequence alignment of DDX56 orthologs from human (GenBank accession number: NP_061955), mice (GenBank accession number: NP_080814), zebrafish (GenBank accession number: NP_001003876), and grass carp (GenBank accession number: XP_051766388) revealed two distinct structural features specific to the fish homologs (Fig. S1C). First, within the conserved Helicase_C domain—a region critical for ATP-dependent RNA unwinding activity—fish DDX56 contains an 8-amino-acid insertion that is absent in mammalian counterparts. Second, a 7-amino-acid insertion (in grass carp) or 8-amino-acid insertion (in zebrafish) was identified at the C-terminal region, a domain often involved in protein-protein interactions and functional modulation of helicases. These lineage-specific insertions may contribute to functional divergence of DDX56 between teleosts and mammals, potentially altering substrate specificity, regulatory interactions, or subcellular localization in the context of antiviral immunity.

**Fig 1:**
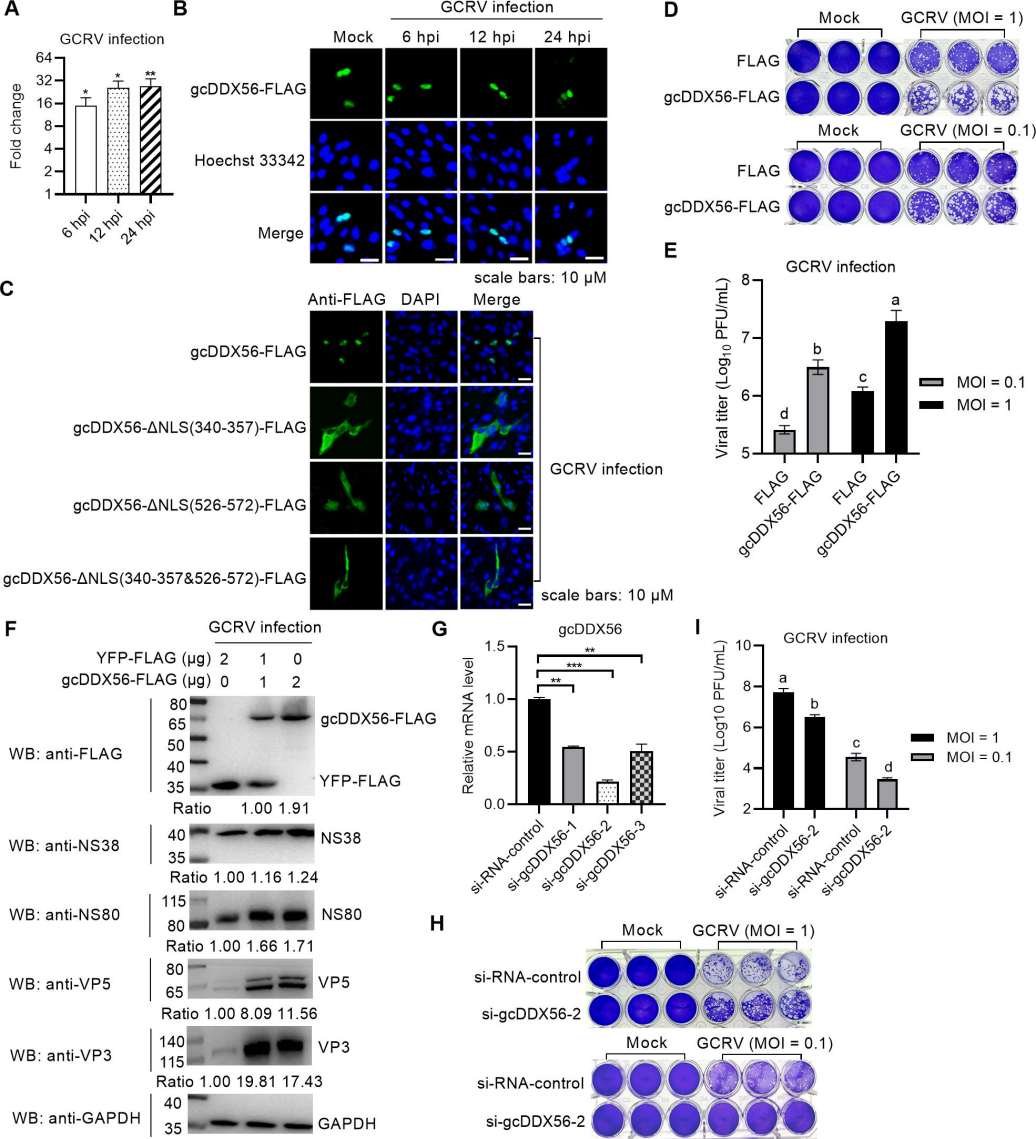
gcDDX56 exhibits infection-induced expression, specific subcellular localization, and pro-viral activity in GCRV-infected CIK cells. (**A**) qRT-PCR analysis of gcDDX56 expression in CIK cells at 6, 12, and 24 hpi with GCRV infection. Statistical significance relative to uninfected controls is indicated: *P < 0.05, **P < 0.01, ***P < 0.001. (**B**) Subcellular localization of gcDDX56 in GCRV-infected CIK cells (MOI = 1). (**C**) Subcellular localization of gcDDX56 NLS motif deletion mutants in GCRV-infected CIK cells (MOI = 1). Scale bars = 10 µM. (**D, E**) Crystal violet staining (**D**) and viral titer quantification (**E**) showing enhanced GCRV replication in CIK cells overexpressing gcDDX56, following GCRV infection at MOI = 0.1 or 1. (**F**) The effect of gcDDX56 overexpression on the expressions of GCRV proteins including NS38, NS80, VP3, and VP5 in CIK cells infected with GCRV at the MOI of 1. Protein bands were quantified by Image J. (**G**) The effect of gcDDX56 knockdown on the expression of gcDDX56 mRNA in CIK cells. CIK cells transfected with si-gcOSBP1-1, si-gcOSBP1-2, or si-gcOSBP1-3 were collected and used for qRT-PCR. (**H, I**) Crystal violet staining (**H**) and viral titer quantification (**I**) demonstrating reduced GCRV replication in CIK cells with gcDDX56 knockdown, following infection at MOI = 0.1 or 1. For (**E, I**), multiple comparisons were performed using ANOVA; different letter superscripts indicate significant differences (a > b > c >d). Error bars represent ± SEM.

Functional characterization of gcDDX56 was performed via GCRV infection studies. qRT-PCR analysis revealed significant upregulation of gcDDX56 transcripts in CIK cells following GCRV infection at an MOI of 1. Specifically, expression levels at 6, 12, and 24 h post-infection (hpi) were all significantly elevated compared to unstimulated controls, with a peak induction of ~27.19-fold observed at 24 hpi (Fig. 1A). Immunofluorescence microscopy further showed that gcDDX56 localized exclusively in the nucleus under both basal and GCRV-infected conditions. Notably, no changes in subcellular localization pattern were observed at any time point examined (6, 12, 24 hpi) (Fig. 1B).

Sequence analysis of gcDDX56 identified two putative nuclear localization signals (NLSs): one within the Helicase C domain (amino acids 340–357) and another in the C-terminal region (amino acids 526–572). To investigate their functional relevance, we generated FLAG-tagged expression plasmids harboring single or double deletions of these NLSs: gcDDX56-ΔNLS(340–357)-FLAG, gcDDX56-ΔNLS(526–572)-FLAG, and gcDDX56-ΔNLS(340–357&526–572)-FLAG. Immunofluorescence microscopy revealed that disruption of both NLSs profoundly impaired the nuclear localization of gcDDX56 (Fig. 1C). Specifically, mutants lacking either the Helicase C domain NLS (340–357) or the C-terminal NLS (526–572), as well as the double deletion mutant, exhibited exclusive cytoplasmic localization, indicating that both NLSs are collectively required for gcDDX56 nuclear import.

### gcDDX56 promotes GCRV infection and replication

To characterize the functional role of gcDDX56 in GCRV pathogenesis, CIK cells were transfected with either empty FLAG vector or gcDDX56-FLAG expression constructs, followed by GCRV infection at multiplicities of infection (MOI) of 0.1 and 1. Crystal violet staining-based cell viability assays revealed that gcDDX56 overexpression significantly reduced cell survival compared to vector controls at both MOI values (Fig. 1D). Viral titer quantification by TCID_50_ assay demonstrated dose-dependent increases in GCRV titers in gcDDX56-overexpressing cells, with statistical significance at both MOIs tested (Fig. 1E). Western blot analysis further confirmed upregulation of viral structural proteins VP3 and VP5, as well as non-structural proteins NS80 and NS38, in a gcDDX56 expression-dependent manner (Fig. 1F).

To further confirm these findings, RNA interference-mediated knockdown of gcDDX56 was performed using three validated siRNA constructs. qRT-PCR screening identified si-gcDDX56-2 as the most effective silencing reagent at 100 nM concentration (Fig. 1G). Functional validation revealed that gcDDX56 knockdown resulted in dose-dependent inhibition of GCRV infection, as measured by both crystal violet staining (Fig. 1H) and viral titration (Fig. 1I).

Collectively, these data establish gcDDX56 as a critical host factor that promotes GCRV replication and cytopathic effect.

### No interaction existed between gcDDX56 and GCRV proteins

**Fig 2:**
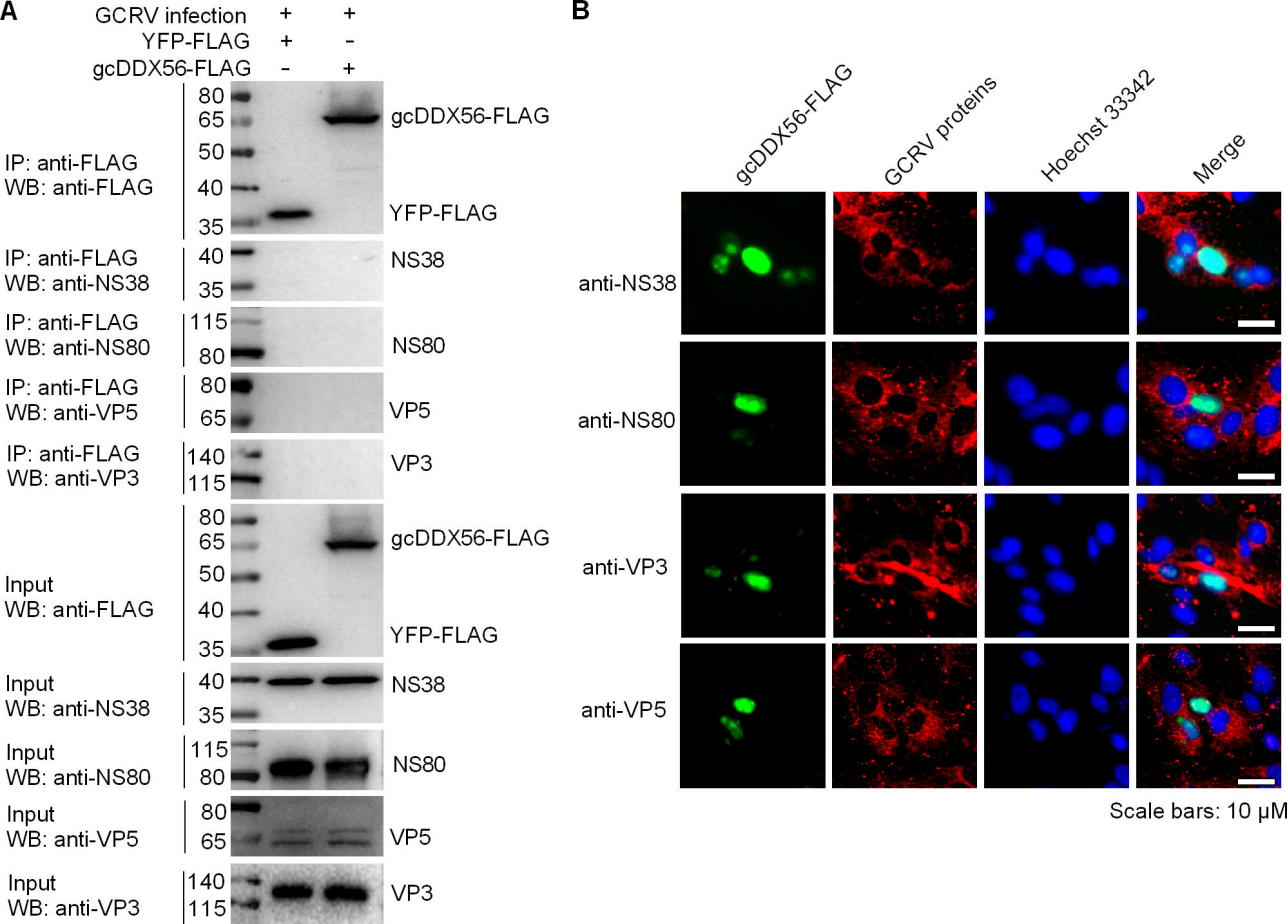
Absence of interactions and colocalization between gcDDX56 and GCRV proteins (NS38, NS80, VP3, VP5). (**A**) No interactions existed between gcDDX56 and GCRV proteins including NS38, NS80, VP3, and VP5. (**B**) No colocalizations existed between gcDDX56 and GCRV proteins including NS38, NS80, VP3, and VP5.

To determine whether gcDDX56 promotes GCRV replication through direct interaction with viral proteins, co-immunoprecipitation (Co-IP) assays were performed. Among the 12 GCRV proteins, two nonstructural proteins (NS38 and NS80), which form VIBs, and two structural proteins (VP3 and VP5) were selected for further investigation. This selection was based on the availability of antibodies against VP3, VP5, NS80, and NS38 (25, 26). CIK cells were transfected with either empty YFG-FLAG vector (negative control) or gcDDX56-FLAG expression construct, followed by GCRV infection. FLAG-tagged gcDDX56 was immunoprecipitated using anti-FLAG M2 affinity resin, and subsequent Western blot analysis detected no co-precipitation of viral proteins VP3, VP5, NS38, or NS80 (Fig. 2A). Immunofluorescence microscopy further supported these findings, showing that GCRV structural proteins VP3/VP5 and non-structural proteins NS38/NS80 displayed punctate cytoplasmic localization following infection. Notably, no co-localization was observed between these viral proteins and gcDDX56 in GCRV-infected cells (Fig. 2B). Collectively, these results indicate that gcDDX56 facilitates GCRV replication through indirect mechanisms rather than direct protein-protein interactions with viral components.

### gcDDX56 inhibits GCRV-triggered activation of IFN1 and IFN3

**Fig 3:**
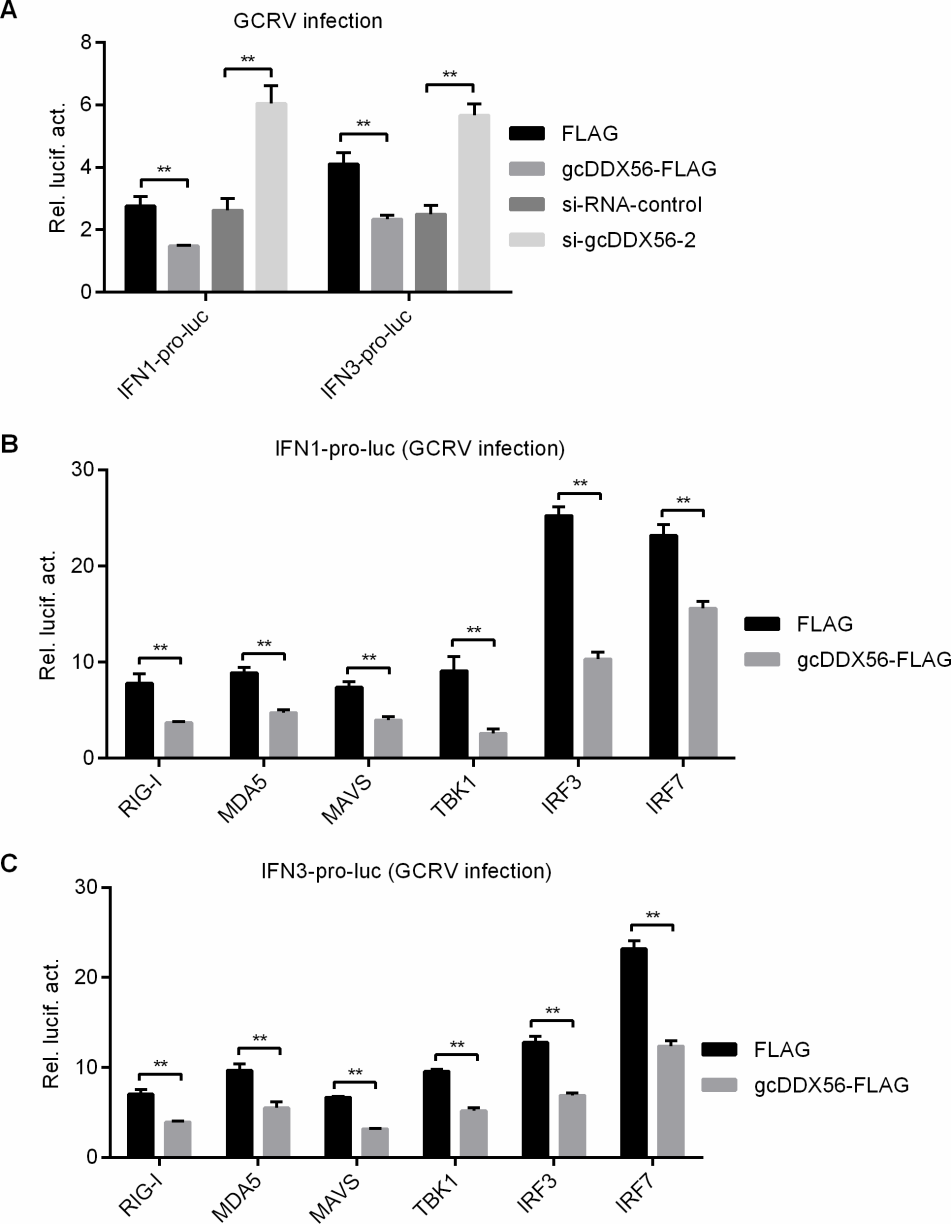
gcDDX56 modulates IFN1 and IFN3 promoter activity via the RLR signaling pathway in GCRV-infected CIK cells. (**A**) The effect of gcDDX56 overexpression or knockdown on the IFN1pro-luc and IFN3pro-luc promoters in CIK cells infected with GCRV (MOI = 1). (**B** and **C**) The effect of gcDDX56 on the IFN1pro-luc (**B**) or IFN3pro-luc (**C**) promoter mediated by RLR signaling pathway in CIK cells infected with GCRV (MOI = 1). (**A–C**) The asterisk indicated statistical significance between the two groups connected by the bracket. **P < 0.01.

In vertebrates, the RLR-mediated signaling pathway plays a pivotal role in recognizing RNA viruses and initiating antiviral immune responses, with type I IFN production serving as an effector of antiviral defense. Previous studies have established that grass carp IFN1 and IFN3 are the principal functional type I IFNs (27). Given the well-documented regulatory roles of DDX helicase family members in type I IFN pathways (28, 29), we sought to characterize the mechanistic basis of gcDDX56’s pro-viral function during GCRV infection. Using a dual-luciferase reporter assay, we assessed the impact of gcDDX56 on IFN1/IFN3 promoter activities during GCRV infection. Overexpression of gcDDX56 significantly attenuated both IFN1 and IFN3 promoter activities, whereas gcDDX56 knockdown via siRNA transfection resulted in a marked augmentation of IFN1/IFN3 promoter activities (Fig. 3A). To dissect the molecular mechanism, we evaluated the effect of gcDDX56 on RLR signaling key components, including upstream PRRs (MDA5, RIG-I), downstream signaling adaptor protein (MAVS), critical kinase (TBK1), and transcription factors (IRF3, IRF7). Co-expression studies revealed that gcDDX56 potently suppressed the IFN1/IFN3 promoter activation induced by these RLR pathway components (Fig. 3B and C). These results collectively demonstrate that gcDDX56 acts as a negative regulator of the RLR signaling axis, thereby dampening type I IFN production and facilitating viral replication.

### gcDDX56 inhibits the expression of gcIRF3 via the autophagy–lysosome pathway

To characterize the molecular mechanism underlying gcDDX56’s regulation of type I IFN production, we focused on IRF3, a critical transcription factor in the RLR signaling axis. Accumulating evidence has established that IRF3 phosphorylation represents a key checkpoint in antiviral innate immunity, particularly in response to RNA virus infections (30, 31). Upon viral recognition, cytoplasmic RIG-I transmits signals through MAVS and TBK1, culminating in IRF3 phosphorylation, nuclear translocation, and subsequent type I IFN transcription.

**Fig 4:**
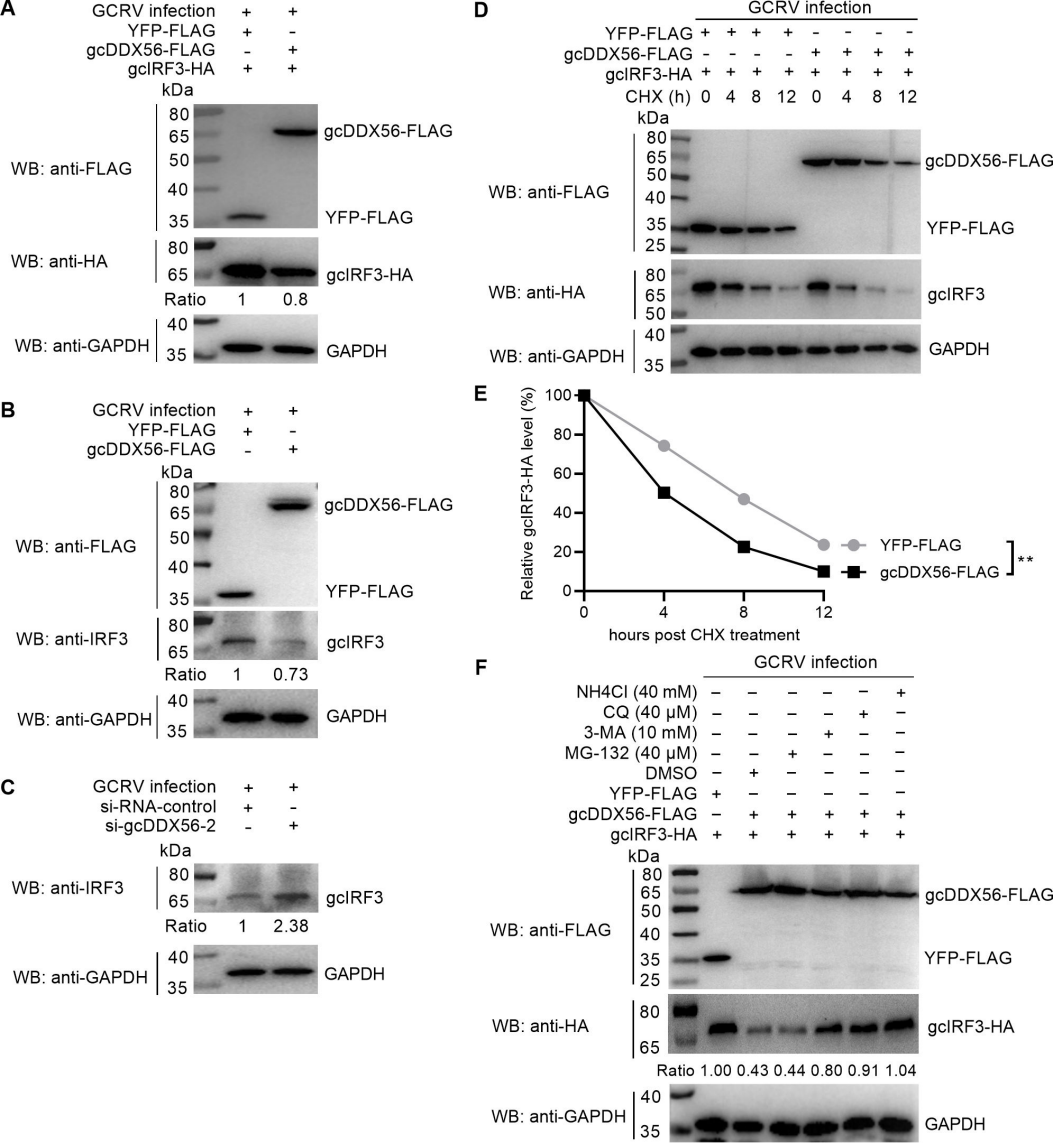
gcDDX56-mediated IRF3 degradation via autophagy–lysosome pathway. (**A**) The effect of gcDDX56 overexpression on the exogenous expression of IRF3. (**B**) The effect of gcDDX56 overexpression on the endogenous expression of IRF3. (**C**) The effect of gcDDX56 knockdown on the endogenous expression of IRF3. (**A–C**) Protein bands were quantified by Image J. (**D**) The effect of gcDDX56 on the IRF3 protein after CHX treatment. (**E**) Relative gcIRF3 protein levels normalized to the housekeeping protein GAPDH. (**F**) gcDDX56 promoted the degradation of IRF3 via autophagosome-lysosome pathway. **P < 0.01.

To determine whether gcDDX56 modulates GCRV replication through IRF3 regulation, co-transfection experiments were performed in CIK cells using gcDDX56-FLAG and IRF3-HA constructs. Western blot analysis revealed that gcDDX56 overexpression significantly reduced gcIRF3 protein abundance following GCRV infection compared to YFP-FLAG controls (Fig. 4A). Endogenous IRF3 detection confirmed these findings, and conversely, gcDDX56 knockdown led to a 2.38-fold increase in gcIRF3 abundance (Fig. 4B and C).

Cycloheximide (CHX) chase assays were utilized to determine whether gcDDX56 affects gcIRF3 protein stability. Overexpression of gcDDX56 markedly degraded gcIRF3 protein in the presence of CHX, an inhibitor of protein synthesis (Fig. 4D and E). Pharmacological inhibitor studies further dissected the degradation pathway: proteasome inhibition with MG132 failed to rescue gcIRF3 levels, whereas autophagy–lysosome inhibitors NH_4_Cl, CQ, and 3-MA significantly restored gcIRF3 protein abundance in gcDDX56-overexpressing cells (Fig. 4F).

Collectively, these results demonstrate that gcDDX56 promotes GCRV replication by targeting gcIRF3 for degradation via the autophagy–lysosome pathway, thereby dampening antiviral type I IFN responses.

### gcDDX56 interacts with gcKPNB3 via its Helicase C domain, mediated by the KAP95 domain of gcKPNB3

**Fig 5:**
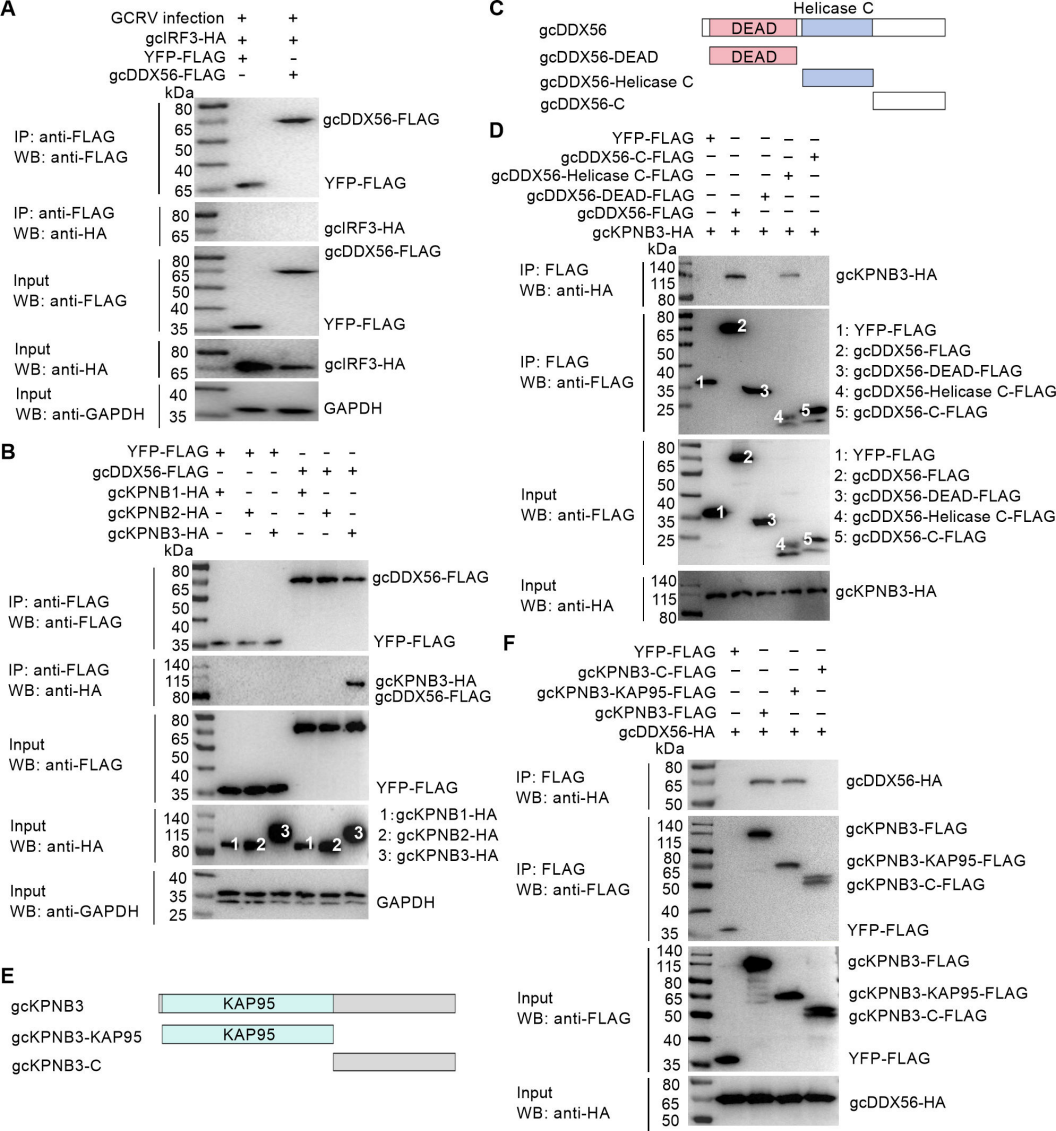
Domain-specific interaction between gcDDX56 and gcKPNB3. (**A**) gcDDX56 does not interact with IRF3 in GCRV-infected cells. (**B**) gcDDX56 interacts with gcKPNB3. (**C**) Domain structure of gcDDX56 and schematic of truncated mutants (gcDDX56-DEAD, gcDDX56-Helicase C, gcDDX56-C). (**D**) Co-IP assay examining interactions between gcKPNB3-HA and gcDDX56-FLAG or its truncated mutants (gcDDX56-DEAD-FLAG, gcDDX56-Helicase C-FLAG, gcDDX56-C-FLAG); YFP-FLAG served as control. Cell lysates and anti-FLAG immunoprecipitates were analyzed by immunoblotting with indicated antibodies. (**E**) Domain structure of gcKPNB3 and schematic of truncated mutants (gcKPNB3-KAP95, gcKPNB3-C). (**F**) Co-IP assay examining interactions between gcDDX56-HA and gcKPNB3-FLAG or its truncated mutants (gcKPNB3-KAP95-FLAG, gcKPNB3-C-FLAG); YFP-FLAG served as control. Cell lysates and anti-FLAG immunoprecipitates were analyzed by immunoblotting with indicated antibodies.

To identify potential adaptor molecules involved in gcDDX56’s regulatory function, Co-IP assays were performed in CIK cells co-transfected with gcDDX56-FLAG and gcIRF3-HA constructs. No direct physical interaction was detected between gcDDX56 and gcIRF3 under infection conditions (Fig. 5A), suggesting an indirect regulatory mechanism. Given the established role of KPNB family members in mediating nuclear import of transcription factors like IRF3, we systematically evaluated interactions between gcDDX56 and grass carp KPNB isoforms (gcKPNB1, gcKPNB2, gcKPNB3). While gcKPNB1, gcKPNB2, and gcKPNB3 each harbor a conserved KAP95 domain, they exhibit substantial sequence divergence, with amino acid sequence identities ranging from 12.9% to 15.4% (Fig. S2). Co-IP analysis revealed specific interaction between gcDDX56 and gcKPNB3 (Fig. 5B).

To map the functional domains of gcDDX56 involved in binding gcKPNB3, three truncated mutants were generated: gcDDX56-DEAD (containing only the DEAD domain), gcDDX56-Helicase C (retaining the Helicase C domain), and gcDDX56-C (encompassing the C-terminal region) (Fig. 5C). Co-IP showed that only gcDDX56-Helicase C, like full-length gcDDX56, bound to gcKPNB3 (Fig. 5D), indicating the Helicase C domain is critical for this interaction. To define the interacting domain in gcKPNB3, two mutants were constructed: gcKPNB3-KAP95 (retaining the KAP95 domain) and gcKPNB3-C (lacking the KAP95 domain, encompassing the C-terminal region) (Fig. 5E). Co-IP demonstrated that the KAP95 domain mediates gcKPNB3-gcDDX56 binding, as gcKPNB3-C lost this interaction (Fig. 5F).

Together, these findings establish a specific, isoform-selective interaction between gcDDX56 and gcKPNB, mediated by the Helicase C domain of gcDDX56 and the KAP95 domain of gcKPNB3, uncovering a novel importin β-dependent mechanism through which gcDDX56 regulates IRF3.

### gcDDX56’s Helicase C domain and gcKPNB3’s KAP95 domain as key for their nuclear co-localization, gcKPNB3 nuclear aggregation, and gcDDX56-regulated gcKPNB3 nucleocytoplasmic shuttling

**Fig 6:**
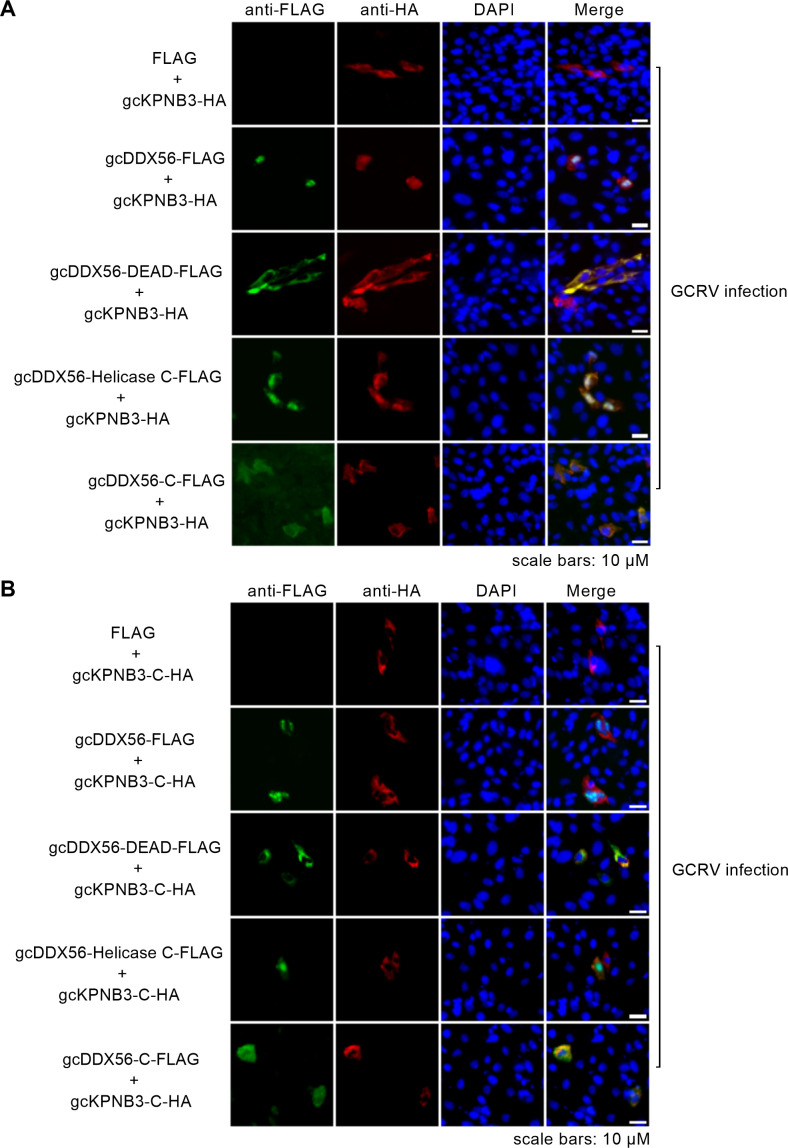
gcDDX56 and its domain mutants regulate subcellular localization of gcKPNB3 and gcKPNB3-ΔKAP95 during GCRV infection. (**A**) Effect of gcDDX56, gcDDX56-DEAD, gcDDX56-Helicase C, and gcDDX56-C on subcellular localization of gcKPNB3 in GCRV-infected cells (MOI = 1). Scale bars = 10 µM. (**B**) Effect of gcDDX56, gcDDX56-DEAD, gcDDX56-Helicase C, and gcDDX56-C on subcellular localization of gcKPNB3-ΔKAP95 in GCRV-infected cells (MOI = 1). Scale bars = 10 µM.

Immunofluorescence microscopy analyses were next performed to dissect how gcDDX56 and its domain-specific mutants modulate the subcellular localization of gcKPNB3 and its KAP95 domain-deleted variant (gcKPNB3-C). These experiments uncovered a critical functional link: the Helicase C domain of gcDDX56—which contains an NLS (340-357)—is indispensable for the nuclear localization of both gcDDX56 itself and gcKPNB3. By contrast, the gcDDX56-C mutant (which retains an NLS at aa 526-572 but lacks the Helicase C domain) exhibited only minimal nuclear localization; its fluorescence signal was predominantly confined to the cytoplasm, and no appreciable nuclear co-localization with gcKPNB3 was observed. The gcDDX56-DEAD mutant (carrying only the DEAD domain, no Helicase C domain) was restricted entirely to the cytoplasm and similarly failed to co-localize with gcKPNB3 in the nucleus. Notably, co-transfection of the gcDDX56-Helicase C mutant with gcKPNB3 triggered robust nuclear aggregation of gcKPNB3—a phenotype that mirrored the nuclear clustering observed when full-length gcDDX56 was co-expressed with gcKPNB3 (Fig. 6A). In sharp contrast, gcKPNB3 remained diffusely distributed (without nuclear aggregation) when co-transfected with either gcDDX56-DEAD or gcDDX56-C, further validating that the Helicase C domain is required for driving gcKPNB3’s nuclear aggregation (Fig. 6A).

Furthermore, gcKPNB3-C (the KAP95 domain-deleted variant) was primarily localized to the cytoplasm and showed no detectable nuclear co-localization with either full-length gcDDX56 or any of its mutants (Fig. 6B). Together, these observations demonstrate that the nuclear co-localization of gcDDX56 and gcKPNB3 is strictly dependent on two domains: the Helicase C domain of gcDDX56 and the KAP95 domain of gcKPNB3. Complementary knockdown experiments further supported this model: depletion of gcDDX56 significantly increased the immunofluorescence intensity of gcKPNB3 in the cytoplasm while reducing its signal intensity in the nucleus (Fig. S3), validating that gcDDX56 directly governs gcKPNB3’s nucleocytoplasmic shuttling.

Collectively, these data establish that gcDDX56’s Helicase C domain not only mediates its own nuclear localization but also drives gcKPNB3’s nuclear accumulation and aggregation, while gcKPNB3’s KAP95 domain is essential for this interprotein interaction and subsequent nuclear co-localization.

### gcDDX56 sequesters gcKPNB3 to disrupt gcIRF3 nuclear import by inhibiting ternary complex formation

**Fig 7:**
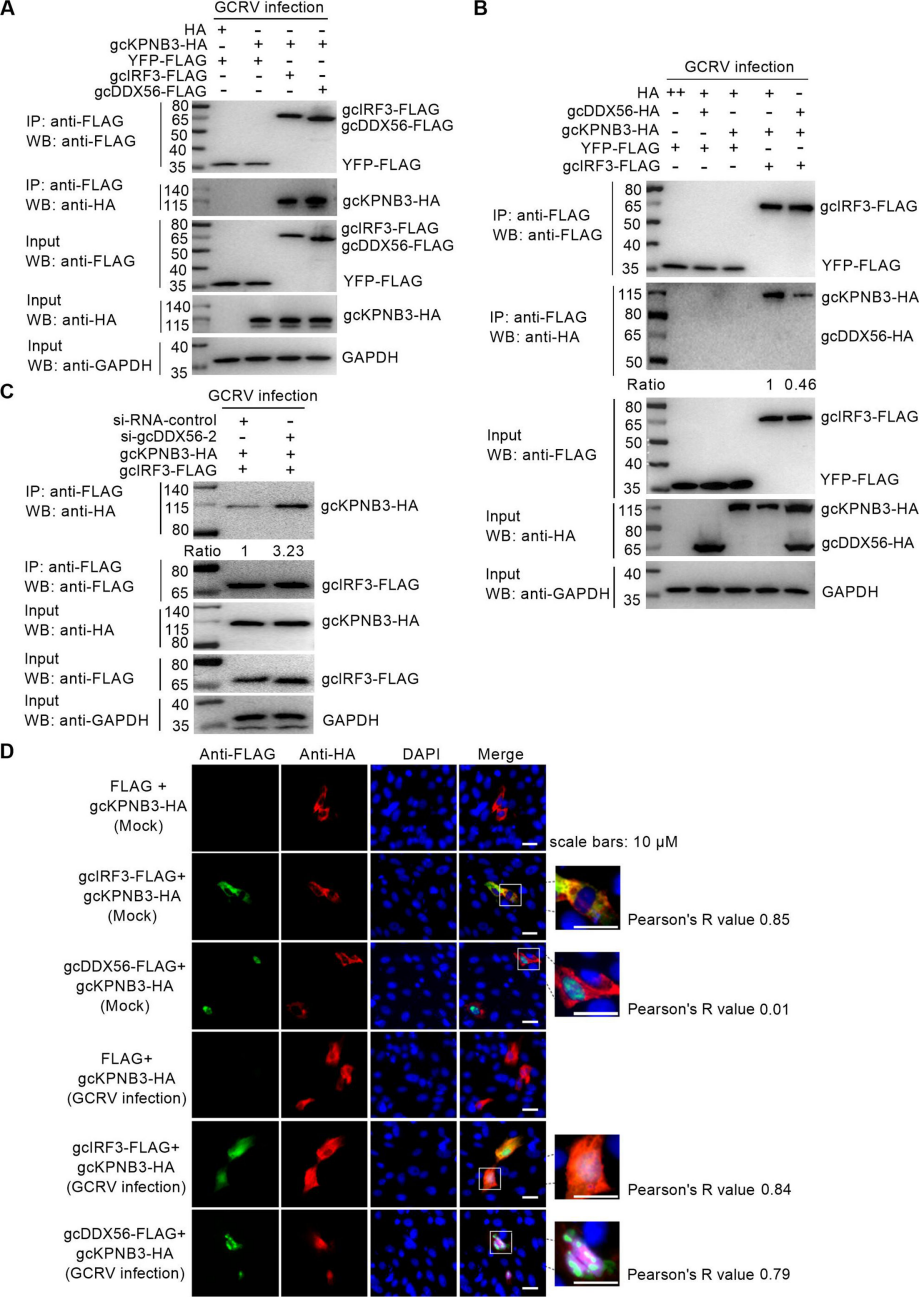
gcDDX56 modulates interactions between gcKPNB3 and IRF3 during GCRV infection. (**A**) gcKPNB3 interacts with IRF3 and gcDDX56. (**B**) gcDDX56 overexpression impairs IRF3-gcKPNB3 complexes in GCRV-infected cells. +, 4 µg; ++, 8 µg. (**C**) gcDDX56 knockdown promotes IRF3-gcKPNB3 complexes in GCRV-infected cells. Protein bands in (**B**) and (**C**) were quantified using ImageJ. (**D**) Subcellular co-localization of gcKPNB3 with IRF3 or gcDDX56 in the nucleus of GCRV-infected cells.

Subsequent pull-down experiments confirmed that gcKPNB3 forms a ternary complex with both gcDDX56 and gcIRF3 during GCRV infection (Fig. 7A). Functional co-expression studies revealed that gcDDX56 overexpression reduced the binding efficiency between gcIRF3 and gcKPNB3 (Fig. 7B), whereas gcDDX56 knockdown increased this interaction by 3.23-fold (Fig. 7C), supporting that gcDDX56 acts as a competitive inhibitor of gcIRF3 nuclear import via gcKPNB3 sequestration.

Immunofluorescence microscopy further characterized the nucleocytoplasmic trafficking dynamics of gcIRF3 and gcKPNB3. In resting cells, gcKPNB3 localized predominantly to the cytoplasm, co-localizing strongly with cytoplasmic gcIRF3 (Pearson’s *R* = 0.85), and showing no overlap with nuclear gcDDX56 (*R* = 0.01). Following GCRV infection, partial pools of both gcIRF3 and gcKPNB3 translocated to the nucleus, maintaining robust co-localization in both compartments (*R* = 0.84), and gcKPNB3 also co-localized significantly with gcDDX56 (*R* = 0.79) (Fig. 7D).

**Fig 8:**
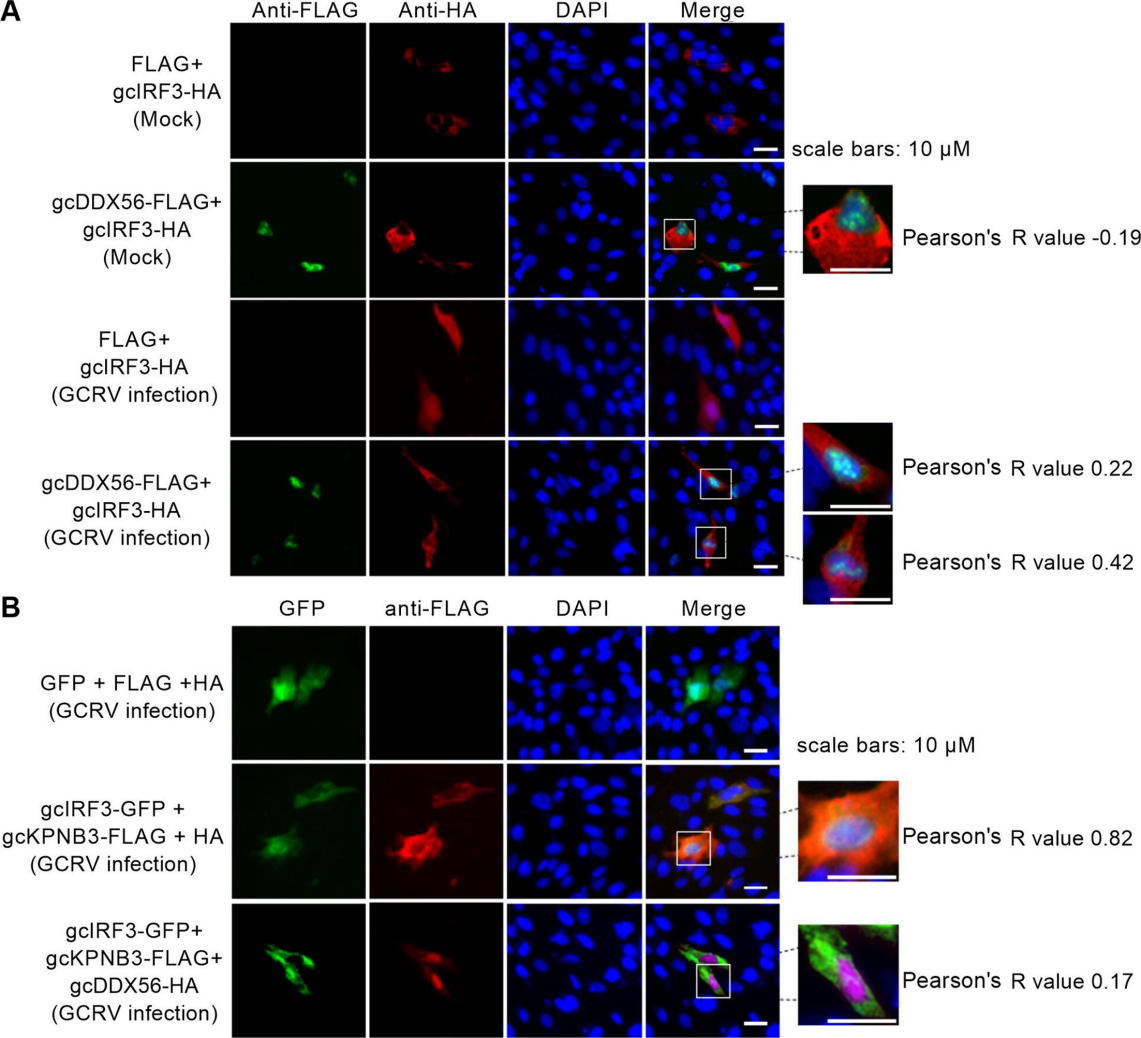
gcDDX56 overexpression alters nuclear fluorescence intensity of IRF3 and gcKPNB3 in GCRV-infected cells. (**A**) gcDDX56 overexpression reduces nuclear IRF3 fluorescence intensity in GCRV-infected cells. (**B**) gcDDX56 overexpression reduces nuclear IRF3 fluorescence intensity while increasing nuclear gcKPNB3 signals in GCRV-infected cells.

In uninfected CIK cells, gcDDX56 was nuclear, and gcIRF3 was cytoplasmic. However, in GCRV-infected cells, co-transfection of gcDDX56-FLAG with gcIRF3-HA significantly reduced nuclear gcIRF3 compared to the control (FLAG+gcIRF3HA) (Fig. 8A), indicating that gcDDX56 modulates gcIRF3 localization during infection. Mechanistic dissection via co-transfection of gcIRF3-GFP, gcKPNB3-FLAG, and gcDDX56-HA showed that gcDDX56 overexpression drastically reduced gcIRF3-gcKPNB3 co-localization (*R* = 0.17 vs control *R* = 0.82) and retained gcIRF3 in the cytoplasm (Fig. 8B).

Together, these data demonstrate that gcDDX56 disrupts gcIRF3 nuclear import by sequestering gcKPNB3, thereby blocking formation of the functional import complex required for IRF3 translocation.

### gcDDX56 modulates gcIRF3 nucleocytoplasmic trafficking and protein stability in a gcKPNB3-dependent manner

To biochemically validate the immunofluorescence observations, nuclear–cytoplasmic fractionation was performed in GCRV-infected CIK cells. Western blot analysis showed that GCRV infection induced nuclear accumulation of gcIRF3 in control cells transfected with empty YFP-FLAG vector, whereas this nuclear translocation was significantly attenuated in cells overexpressing gcDDX56 (Fig. S4A). Conversely, gcDDX56 knockdown resulted in a 1.79-fold increase in nuclear gcIRF3 levels compared to siRNA controls (Fig. S4B), confirming that gcDDX56 suppresses gcIRF3 nuclear translocation during GCRV infection.

**Fig 9:**
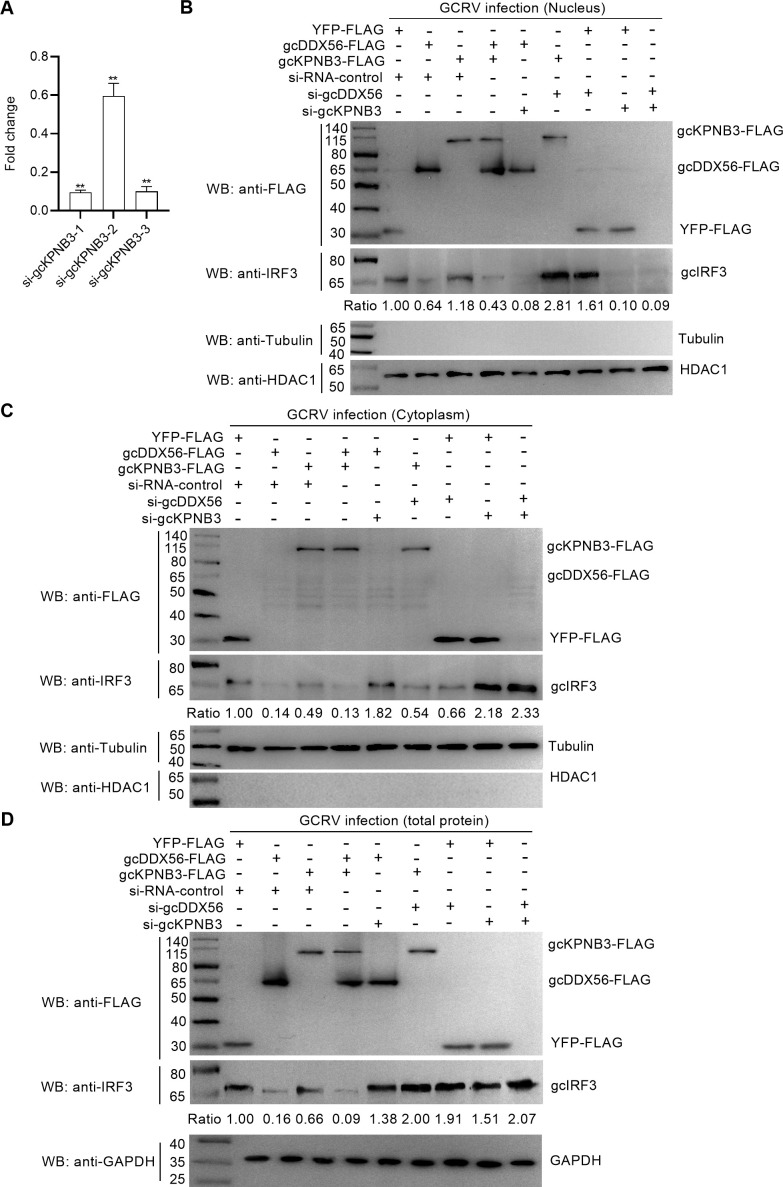
Validation of gcKPNB3 silencing efficiency and subcellular/ total protein level analyses of gcIRF3 mediated by gcDDX56 and/or gcKPNB3. (**A**) qRT-PCR analysis of gcKPNB3 silencing efficiency in CIK cells transfected with si-gcKPNB3-1, −2, or −3. **P < 0.01. (**B**) Nuclear levels of gcIRF3 mediated by gcDDX56 and/or gcKPNB3. (**C**) Cytoplasmic levels of gcIRF3 mediated by gcDDX56 and/or gcKPNB3. (**D**) Total protein levels of endogenous IRF3 mediated by gcDDX56 and/or gcKPNB3 in GCRV-infected cells (MOI = 1) with overexpression and/or knockdown of gcDDX56 and/or gcKPNB3. Tubulin (cytoplasmic) and HDAC1 (nuclear) served as fractionation controls; GAPDH as internal control. Endogenous IRF3 was detected with anti-IRF3 antibodies. Band intensities were quantified via Image J.

To dissect the functional interplay between gcDDX56 and gcKPNB3 in regulating gcIRF3, we first validated siRNA efficacy for gcKPNB3: qRT-PCR results identified si-gcKPNB3-1 (100 nM) as the most effective silencing reagent (Fig. 9A). Subsequent nucleocytoplasmic fractionation assays revealed distinct regulatory patterns:

- Nuclear gcIRF3: Overexpression of gcDDX56 reduced nuclear gcIRF3 levels; gcKPNB3 overexpression alone had no significant effect, but gcKPNB3 knockdown further enhanced gcDDX56-mediated reduction of nuclear gcIRF3. Conversely, gcDDX56 knockdown increased nuclear gcIRF3, and this effect was amplified by gcKPNB3 overexpression. Notably, gcDDX56 knockdown failed to increase nuclear gcIRF3 when gcKPNB3 was depleted (Fig. 9B).
- Cytoplasmic gcIRF3: Overexpression of either gcDDX56 or gcKPNB3 decreased cytoplasmic gcIRF3, while gcKPNB3 knockdown abolished gcDDX56-induced cytoplasmic gcIRF3 degradation. Intriguingly, gcDDX56 knockdown also reduced cytoplasmic gcIRF3, whereas gcKPNB3 knockdown alone increased it (Fig. 9C). However, gcDDX56 knockdown did not alter the effects of gcKPNB3 knockdown on cytoplasmic or nuclear gcIRF3 (Fig. 9B and C).
- Total gcIRF3: Analysis of total gcIRF3 protein levels showed consistent trends. gcDDX56 or gcKPNB3 overexpression decreased total gcIRF3, and gcKPNB3 knockdown abrogated gcDDX56-mediated total gcIRF3 degradation. In contrast, knockdown of either gcDDX56 or gcKPNB3 increased total gcIRF3, with no synergistic effect observed between the two knockdowns (Fig. 9D).

Collectively, these data demonstrate that gcDDX56 regulates both gcIRF3 nucleocytoplasmic trafficking and protein stability through a mechanism dependent on gcKPNB3, with gcKPNB3 serving as a critical mediator of gcDDX56’s inhibitory effects on gcIRF3 function.

### gcKPNB3 drives gcIRF3 degradation via the autophagy–lysosome pathway

Our prior nucleocytoplasmic fractionation assays established that gcKPNB3 reduces both cytoplasmic and total gcIRF3 protein levels (Fig. 9C and D; Table S2)—an observation that prompted a critical mechanistic question: does this reduction stem from transcriptional repression, or enhanced post-translational degradation? This distinction is biologically critical, as IRF3’s antiviral function is tightly controlled by protein turnover. Given that nucleocytoplasmic fractionation also revealed gcIRF3 subcellular redistribution, we prioritized investigating protein degradation, as changes in subcellular localization often correlate with altered protein turnover (e.g., sequestration in degradative compartments).

To first validate gcKPNB3’s role in regulating gcIRF3 under physiologically relevant conditions (i.e., GCRV infection, where host cells actively upregulate IRF3 to counter viral replication), we performed Western blot analysis in GCRV-infected CIK cells. Results showed that gcKPNB3 overexpression still significantly reduced gcIRF3 protein abundance compared to empty YFP-FLAG vector controls (Fig. S5A), indicating that gcKPNB3 actively suppresses gcIRF3 even under conditions in which the host immune system favors IRF3 accumulation.

We next directly tested whether gcKPNB3 drives gcIRF3 degradation. CIK cells were transfected with gcKPNB3-FLAG or YFP-FLAG and then treated with CHX to block new protein production. Over time, gcKPNB3-overexpressing cells exhibited markedly accelerated gcIRF3 protein loss relative to CHX-treated controls with YFP-FLAG transfection (Fig. S5B and C). This result definitively excluded reduced transcription or translation as the cause of gcIRF3 reduction and confirmed that gcKPNB3 enhances gcIRF3 degradation.

To delineate the degradation pathway, we employed pharmacological inhibitors targeting two major protein degradation systems: the proteasome and the autophagy–lysosome pathway. Treatment with the proteasome inhibitor MG132 failed to rescue gcIRF3 protein levels in gcKPNB3-overexpressing cells (Fig. S5D). In striking contrast, three distinct inhibitors (NH_4_Cl, CQ, and 3-MA) of the autophagy–lysosome pathway restored gcIRF3 abundance (Fig. S5D). The consistency of results across multiple autophagy–lysosome inhibitors unambiguously confirmed that gcKPNB3 mediates gcIRF3 degradation via the autophagy–lysosome axis.

Collectively, these data identify gcKPNB3 as a key mediator of gcIRF3 degradation via the autophagy–lysosome pathway.

### gcDDX56 suppresses IRF3-driven transcriptional activation and promotes GCRV replication in a gcKPNB3-dependent manner

**Fig 10:**
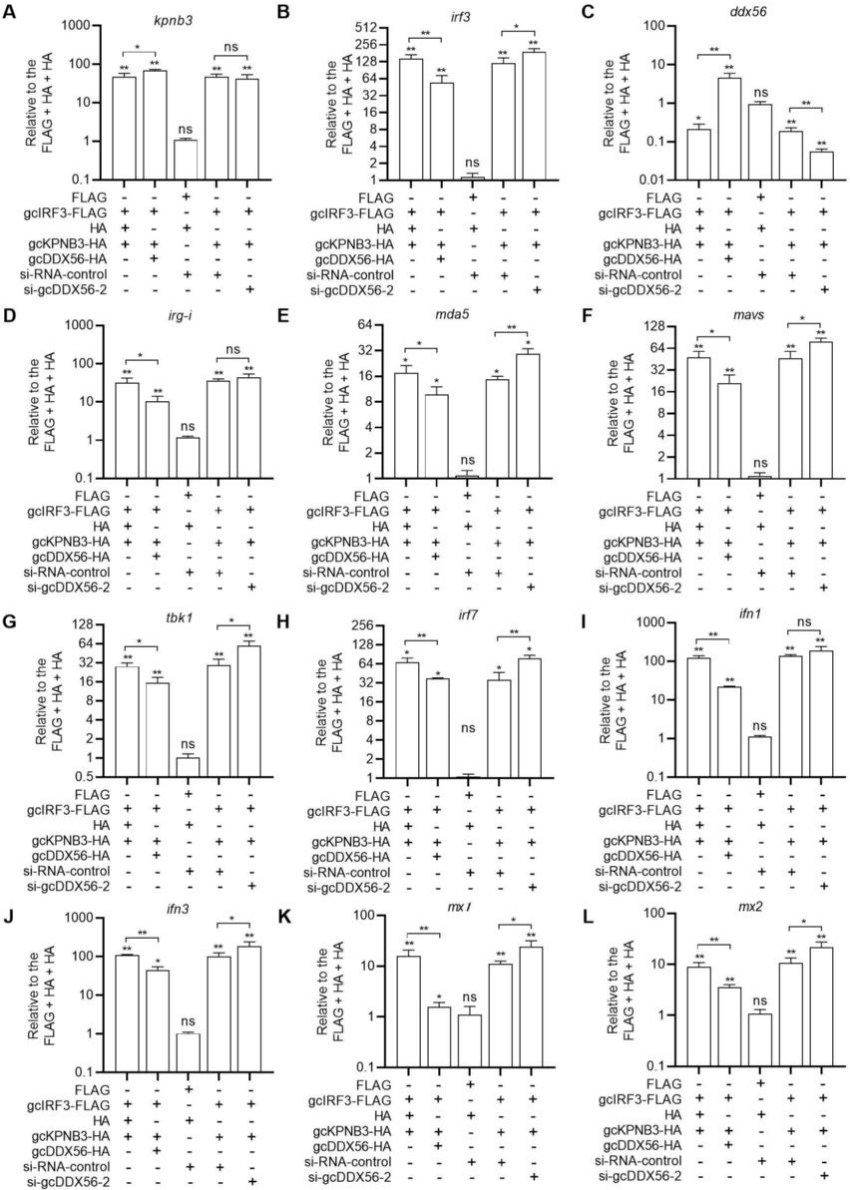
The effects of gcDDX56 overexpression or knockdown on the transcription of *kpnb3* (**A**), *irf3* (**B**), *gcddx56* (**C**), *rig-i* (**D**), *mda5* (**E**), *mavs* (**F**), *tbk1* (**G**), *irf7* (**H**), *ifn1* (**I**), *ifn3* (**J**), *mx1* (**K**), and *mx2* (**L**) induced by gcIRF3-gcKPNB3 complexes. The asterisk above the error bars indicated statistical significance using the group transfected with empty plasmids as the control group. The asterisk above the bracket indicated statistical significance between the two groups connected by the bracket. *P < 0.05; **P < 0.01; ns, not significant.

To assess the transcriptional consequences of gcDDX56-mediated gcIRF3 sequestration and degradation, qRT-PCR was performed in CIK cells co-transfected with gcIRF3, gcKPNB3, and gcDDX56 constructs, with cells transfected with empty vectors (FLAG + HA + HA) as controls. Co-expression of gcIRF3 and gcKPNB3 significantly increased the transcript levels of both *gcirf3* and *gckpnb3* (Fig. 10A and B). In contrast, gcIRF3/gcKPNB3 co-expression suppressed *gcddx56* mRNA levels. This suppression was rescued by gcDDX56 overexpression and further enhanced by gcDDX56 knockout (Fig. 10C).

Analysis of RLR signaling components revealed that gcIRF3/gcKPNB3 co-expression robustly activated transcription of pattern recognition receptors (*rig-i*: 31.88-fold; *mda5*: 17.57-fold), the signaling adaptor *mavs* (47.58-fold), the kinase *tbk1* (28.12-fold), the transcription factor *irf7* (68.11-fold), and type I IFNs (*ifn1*: 122.13-fold; *ifn3*: 109.67-fold). These inductive effects were blunted by gcDDX56 overexpression and potentiated by gcDDX56 knockdown (Fig. 10D through 10J). Similarly, gcIRF3/gcKPNB3-induced transcription of ISGs *mx1* (15.81-fold) and *mx2* (8.96-fold) was significantly suppressed by gcDDX56 overexpression, whereas gcDDX56 knockdown augmented *mx1*/*mx2* transcript levels to 24.31-fold and 21.86-fold of control, respectively (Fig. 10K and L).

**Fig 11:**
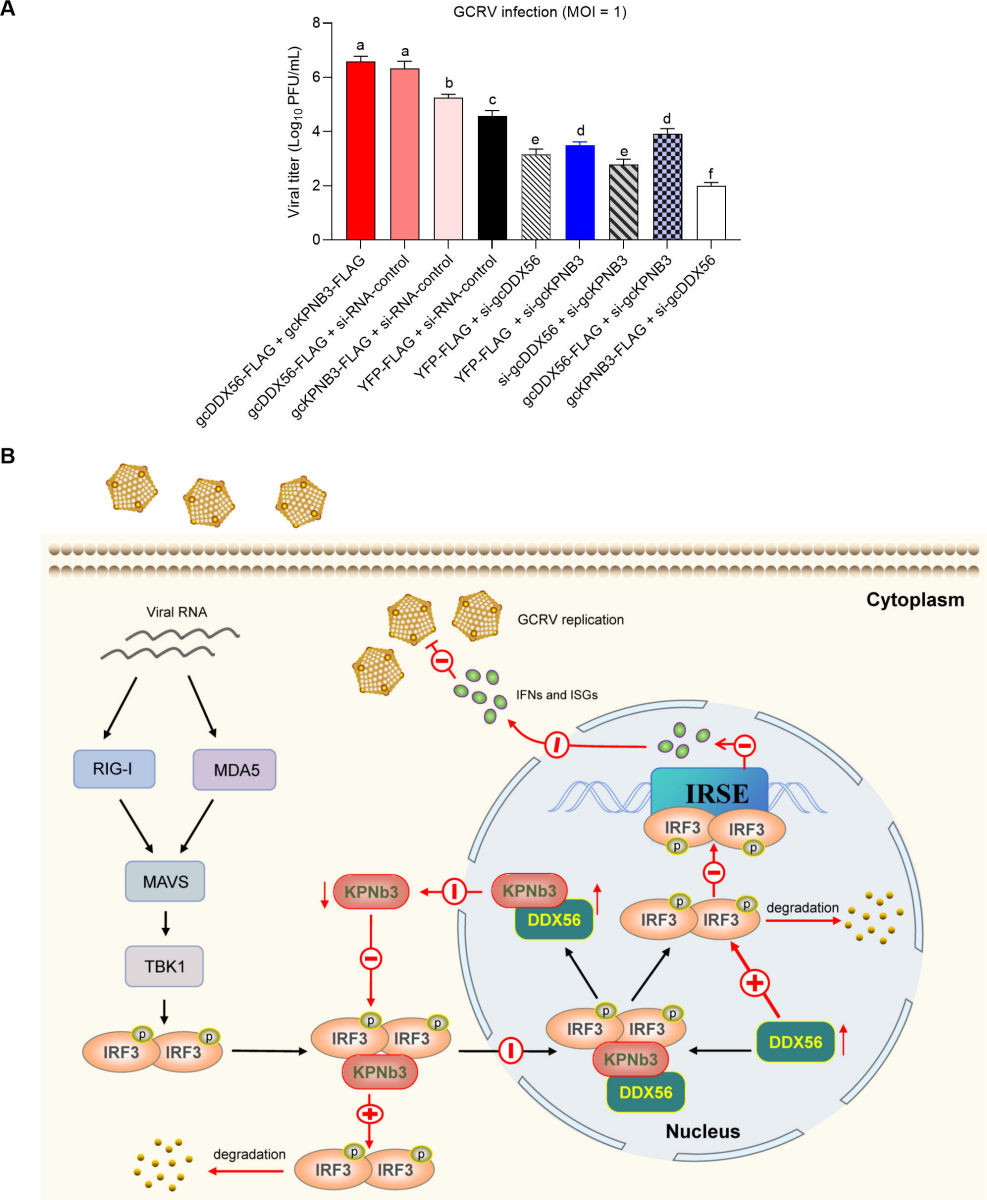
Viral titer analysis and proposed pro-viral mechanism of gcDDX56. (**A**) Quantification of GCRV viral titers in cells with overexpression and/or knockdown of gcDDX56 and/or gcKPNB3 (infected with GCRV at MOI = 1). Multiple comparisons by ANOVA; different letter superscripts indicate significant differences. Error bars: ±SEM. (**B**) Proposed model of gcDDX56’s pro-viral mechanism during GCRV infection. gcDDX56 specifically interacts with the KAP95 domain of gcKPNB3 (an importin β with dual IRF3-transporting and -degrading functions) via its Helicase C domain, acting as a “functional switch” to tilt gcKPNB3 toward cytoplasmic IRF3 degradation while sequestering gcKPNB3 in the nucleus to block IRF3 nuclear import. Concurrently, gcDDX56 promotes autophagy-dependent degradation of nuclear IRF3, collectively forming a “triple inhibitory network” (cytoplasmic IRF3 degradation + nuclear IRF3 clearance + IRF3 import blockade) that suppresses IFN-mediated antiviral signaling and thereby facilitates GCRV replication. Symbols: “⊕” (promotion), “⊝” (inhibition).

We further evaluated the impact of gcDDX56/gcKPNB3 overexpression or knockdown on GCRV replication (Fig. 11A; Table S2). Overexpression of gcDDX56, gcKPNB3, or their combination reduced total IRF3 protein levels (Fig. 9D) and enhanced GCRV replication (Fig. 11A). Conversely, knockdown of gcDDX56, gcKPNB3, or both increased total IRF3 (Fig. 9D) and suppressed viral replication (Fig. 11A). Functional interplay analyses revealed mutual dependence: knockdown of gcKPNB3 abrogated gcDDX56-mediated viral promotion, while gcDDX56 knockdown similarly impaired gcKPNB3’s pro-viral activity.

Collectively, these findings reflect a hierarchical, context-dependent functional interplay between gcDDX56 and gcKPNB3, rooted in their mechanistic coordination in regulating IRF3.

## DISCUSSION

RNA helicases in eukaryotes are classified into six distinct families based on conserved sequence motifs, including DEAD-box, DEAH/RHA, Ski2-like, Upf1-like, RIG-I-like, and NS3/NPH-II (32). Among these, Dead-box proteins play evolutionarily conserved roles in fundamental RNA metabolic processes, including transcription, pre-mRNA splicing, ribosome biogenesis, and translational regulation (33). In mammals, DDX56 belonging to the DEAD-box family not only regulates RNA metabolism but also modulates immune and inflammatory responses. Mammalian DDX56 interacts with viral proteins to facilitate replication of pathogens, such as Influenza A viruses (IAV) and FMDV (24, 34). Despite these advances, the role of DDX56 in teleost antiviral immunity remains poorly characterized. Here, we uncovered a novel role for gcDDX56 in negative regulation of host antiviral responses. GCRV infection induced upregulation of *gcddx56* in CIK cells, which in turn promoted viral replication. Mechanistically, gcDDX56 exerted dual inhibitory effects on the RLR signaling axis: post-translational degradation of IRF3 and disruption of nuclear import machinery. This study establishes for the first time a causal relationship between gcDDX56, the gcIRF3/gcKPNB3 nuclear import complex, and GCRV pathogenesis.

Viruses commandeer host cellular machinery and co-opt host proteins to facilitate their replication, while hosts deploy an arsenal of antiviral factors to counteract infection. Among these, DEAD-box RNA helicases emerge as versatile players in both viral propagation and host defense. Similar to other members of the DEAD-box helicase family, DDX56 has been documented to play dual roles in viral pathogenesis. Mechanistic studies have shown that DDX56 promotes the infectivity or replication of viruses for West Nile virus (WNV), FMDV, and IAV (24, 34, 35). Conversely, overexpression of DDX56 exerts antiviral effects against chikungunya virus (CHIKV) through direct binding to viral genomic RNA in the cytoplasm, leading to genome destabilization and loss of infectivity (36). Our study identifies gcDDX56 as a novel pro-viral factor in GCRV pathogenesis. Unlike CHIKV-induced cytoplasmic translocation of human DDX56, whose localization affected its activity on CHIKV RNA (36), gcDDX56 retained nuclear localization throughout infection, suggesting a species-specific regulatory mechanism. Furthermore, Co-IP assays also demonstrated a notable divergence of gcDDX56 from mammalian DDX56: human DDX56 could bind viral RNA or interact with capsid protein and nonstructural proteins (34–36), while the absence of direct interaction between gcDDX56 and GCRV structural and nonstructural proteins localized in the cytoplasm. Despite the lack of direct interaction with viral proteins, gcDDX56 overexpression augmented the accumulation of GCRV structural proteins VP3 and VP5, as well as non-structural proteins NS38 and NS80. This suggests the involvement of host co-factors, potentially via nuclear transport pathways.

The recognition of viral pathogens by innate immune cells is orchestrated by multiple families of PRRs (37). These diverse PRR systems initiate complex signaling networks that converge on the activation of transcription factors NF-κB and IRF3/7 (38). In enterovirus (EMCV) infection, DDX56 directly binds KPNA3/KPNA4, which are critical nuclear import receptors. This interaction prevents IRF3 phosphorylation and nuclear translocation, subsequently leading to the blockade of IFN-β production (39). While mammalian DDX56 directly interacts with IRF3 to block its nuclear translocation during viral infection (40), our study reveals a distinct regulatory mechanism in teleost fish. Functional analysis demonstrated that gcDDX56 does not directly bind gcIRF3, suggesting an alternative pathway involving intermediate adaptor proteins. Given the critical role of importin α/β-mediated nuclear transport in antiviral signaling (16), we characterized the interaction network of gcDDX56 with nuclear import machinery. Co-IP assays demonstrated that gcDDX56 specifically interacted with gcKPNB3 following GCRV infection. Concurrently, gcIRF3 was found to form a functional complex with gcKPNB3, which was competitively disrupted by gcDDX56 overexpression. This competitive binding suggests a molecular decoy mechanism, where gcDDX56 sequesters gcKPNB3 to impair gcIRF3 nuclear import. Confocal microscopy revealed significant nuclear co-localization of gcIRF3-gcKPNB3 complexes in GCRV-infected cells, in addition to cytoplasmic interactions. Strikingly, gcDDX56 exhibited specific nuclear co-localization with gcKPNB3 post-infection. Overexpression of gcDDX56 resulted in the reduction of nuclear IRF3 fluorescence intensity, concurrent with enhanced nuclear gcKPNB3 signals. This suggests that gcDDX56 disrupts the gcIRF3-gcKPNB3 interaction by sequestering gcKPNB3 in the nucleus, thereby blocking IRF3 nuclear translocation (Fig. 11B).

Our findings further demonstrate that gcDDX56 specifically engages gcKPNB3 via its Helicase C domain, with this interaction mediated by the KAP95 domain of gcKPNB3—a conserved HEAT-repeat region typical of importin β family members. This domain specificity aligns with the well-characterized role of HEAT repeats in importin β proteins, which govern cargo recognition through both classical and non-classical NLS motifs (41, 42). While our data confirm a direct physical interaction between these domains, the precise molecular interface remains undefined: gcDDX56 may either bind to the cargo-binding pocket within gcKPNB3’s HEAT repeats (thereby competing with gcIRF3) or allosterically modulate gcKPNB3 conformation to disrupt its ability to engage cargo. Resolving this distinction will require structural analyses—such as cryo-electron microscopy or X-ray crystallography of the gcDDX56-Helicase C/gcKPNB3-KAP95 complex—to map contact residues and determine whether gcDDX56 acts as a competitive inhibitor of gcIRF3 binding or induces conformational changes that impair gcKPNB3’s nuclear import function. Notably, the gcDDX56-C mutant (harboring the C-terminal NLS) exhibits limited nuclear localization without concomitant gcKPNB3 co-localization, indicating that the Helicase C domain functions not merely as a nuclear targeting module but as a critical functional interface for gcKPNB3 engagement. This observation hints at a unique regulatory mode: gcDDX56 may act as a “molecular switch” that sequesters gcKPNB3 in the nucleus in a domain-dependent manner, thereby tuning gcIRF3’s access to the nuclear compartment. Future studies employing single-molecule tracking or FRET-based approaches could resolve the dynamic spatiotemporal dynamics of this interaction during GCRV infection, clarifying whether gcDDX56 modulates gcKPNB3 recycling or retention in specific subcellular compartments to exert its regulatory effects.

A hallmark of antiviral innate immunity is IRF3/7 activation, which drives IFNs induction to orchestrate antiviral defense (43). Beyond canonical RNA sensors like RIG-I and MDA5, DEAD-box helicases (e.g., DDX3, DDX41) play pivotal roles in viral nucleic acid recognition and IFN-β induction (5). Among these, DDX56 exhibits context-dependent regulation of type I IFN responses across viruses: it suppresses IFN-β via disrupting IRF3 phosphorylation during FMDV infection (24), blocks IRF3-IPO5 nuclear import (40), and employs IFN-independent mechanisms against CHIKV (36). Against this backdrop, the present study defined how gcDDX56 negatively regulates GCRV infection, with a focus on IRF3-dependent IFN signaling. Functional assays revealed that gcDDX56 overexpression reduces both ectopic and endogenous IRF3 during GCRV infection, while knockdown rescues IRF3 levels. CHX chase assays with autophagy inhibitors (3-MA, CQ, NH_4_Cl) confirm gcDDX56 promotes IRF3 degradation via the autophagy–lysosome pathway. Concomitantly, gcDDX56 knockdown increases nuclear IRF3 (with reduced cytoplasmic pools), indicating it is also involved in IRF3 nuclear translocation. This dual regulation—modulating IRF3 stability and activation—distinguishes gcDDX56’s role in GCRV infection from other viral contexts, highlighting lineage-specific adaptations of DEAD-box helicases in immune modulation.

Notably, gcKPNB3 exhibits complementary yet distinct IRF3 regulation: it predominantly suppresses cytoplasmic IRF3 (without altering nuclear pools) via degradation, while simultaneously mediating IRF3 nuclear import. This dual functionality expands importin β family roles beyond cargo transport to include cytoplasmic proteostasis, acting as a “bifunctional checkpoint” that balances trafficking and protein turnover. Despite mediating IRF3 nuclear import (theoretically antiviral), gcKPNB3 is net pro-viral: its cytoplasmic degradation reduces total IRF3 to override transport benefits, as evidenced by gcKPNB3 knockdown increasing total IRF3 and suppressing GCRV replication.

The present results clearly reveal the functional relevance of gcDDX56 and gcKPNB3. Rather than acting independently, the two proteins jointly regulate host antiviral signaling to influence viral replication through a coordinated mechanism characterized by “gcDDX56 directing functional orientation and gcKPNB3 executing dual effects.” The core logic can be broken down into three key aspects:

- Co-expression of gcDDX56 and gcKPNB3: synergistic enhancement of IRF3 inhibition to create an “optimal environment” for GCRV replication. When co-expressed, nuclear, cytoplasmic, and total IRF3 levels are all downregulated, and GCRV replication is enhanced. This reflects the core of their functional synergy, which relies on the key properties of each protein: gcDDX56 binds to the KAP95 domain of gcKPNB3 via its Helicase C domain, “sequestering” a portion of gcKPNB3 in the nucleus. This not only reduces the total amount of gcKPNB3 available in the cytoplasm to mediate IRF3 nuclear import but also clears the small pool of IRF3 that has entered the nucleus through gcDDX56-dependent degradation of nuclear IRF3. The remaining cytoplasmic gcKPNB3 (not sequestered in the nucleus) retains its inherent activity to degrade cytoplasmic IRF3, further reducing cytoplasmic IRF3 levels. Together, they form a triple inhibitory network consisting of “cytoplasmic IRF3 degradation + nuclear IRF3 clearance + blockage of IRF3 nuclear import.” This leads to a significant reduction in total IRF3, maximally suppressing antiviral signaling (IFN/ISG), and ultimately promoting GCRV replication.
- Overexpression of gcDDX56 + inhibition of gcKPNB3: loss of gcKPNB3 relieves IRF3 total level inhibition, counteracting gcDDX56-mediated downregulation of nuclear IRF3. In this combination, nuclear IRF3 is downregulated, while cytoplasmic and total IRF3 are upregulated, and GCRV replication is suppressed. The core lies in the fact including irreplaceability of gcKPNB3 and functional limitation of gcDDX56 alone. gcKPNB3 is the main mediator of cytoplasmic IRF3 degradation. When inhibited, the degradative pathway for cytoplasmic IRF3 is abrogated, leading to the accumulation of cytoplasmic IRF3 and a subsequent increase in total IRF3. Although overexpressed gcDDX56 can still degrade nuclear IRF3 (resulting in downregulated nuclear IRF3), it cannot compensate for the defective cytoplasmic IRF3 degradation caused by gcKPNB3 inhibition. At this point, the upregulation of total IRF3 takes priority over the local downregulation of nuclear IRF3—the accumulated IRF3 in the cytoplasm can enter the nucleus through other nuclear transport proteins (e.g., other redundant pathways), initiating sufficient antiviral signaling to ultimately suppress GCRV replication.
- Overexpression of gcKPNB3 + inhibition of gcDDX56: loss of gcDDX56 unlocks the “transport-function bias” of gcKPNB3, promoting IRF3 nuclear accumulation. In this combination, both nuclear and total IRF3 are upregulated, and GCRV replication is suppressed. This essentially reflects the disappearance of gcDDX56’s “functional switch” role on gcKPNB3. During normal infection, gcDDX56 binds to gcKPNB3, “locking” its function into cytoplasmic IRF3 degradation (rather than nuclear transport). When gcDDX56 is inhibited, this “functional lock” is released, and overexpressed gcKPNB3 can freely exert its dual properties—“nuclear transport as the main function, degradation as the auxiliary function.” It extensively mediates the entry of cytoplasmic IRF3 into the nucleus, while its degradative function is “diluted by transport function” (more gcKPNB3 is used to bind IRF3 and mediate nuclear transport rather than degrade IRF3). On one hand, gcKPNB3-mediated IRF3 nuclear import is enhanced. On the other hand, the loss of gcDDX56 shuts down the gcDDX56-dependent degradation pathway of nuclear IRF3, increasing the stability of nuclear IRF3. Together, these two effects drive significant nuclear accumulation of IRF3, strongly activating antiviral signaling and ultimately suppressing GCRV replication. The functional relevance of the two proteins during GCRV infection can be summarized as a “guidance-execution” regulatory axis, where gcDDX56 acts as a “functional guidance factor” and gcKPNB3 acts as an “effector executor.” By binding to gcKPNB3, gcDDX56 determines the latter’s functional bias (IRF3 degradation vs IRF3 transport). In the presence of gcDDX56, gcKPNB3 tends to degrade IRF3; in the absence of gcDDX56, gcKPNB3 tends to transport IRF3. With its dual functions of “degradation + transport,” gcKPNB3 directly regulates the total level and nuclear localization of IRF3, ultimately determining the strength of antiviral signaling.

In conclusion, our study establishes that the gcDDX56-gcKPNB3-IRF3 axis represents a critical regulatory node in antiviral immunity, one that GCRV has evolved to exploit. By enhancing gcDDX56 activity (directly or indirectly), the virus co-opts gcKPNB3’s degradation function to deplete cytoplasmic IRF3 while blocking its nuclear transport, together with promoting nuclear IRF3 degradation through the autophagy pathway—three mechanisms that converge to suppress IFN signaling. This strategy is particularly effective because it targets IRF3 at both the protein stability and subcellular localization levels, ensuring robust immune evasion. From an evolutionary perspective, this underscores how viruses leverage host proteins with dual functions to maximize their replication. gcKPNB3’s ability to both transport and degrade IRF3 likely evolved to fine-tune immune responses (preventing overactivation while enabling signaling), but GCRV hijacks this balance via gcDDX56, converting a homeostatic mechanism into a pro-viral one. However, our focus on IRF3 does not preclude the possibility that gcDDX56 targets other IRFs (e.g., IRF7). Furthermore, as a DEAD-box helicase, gcDDX56 is likely activated by either virus-derived pathogen-associated molecular patterns (PAMPs) or host stress signals elicited during GCRV replication. These are non-mutually exclusive mechanisms, and they align with the well-documented functional versatility of DEAD-box helicases: this protein family is known to act not only as sensors of viral RNA but also as stress-responsive regulators that fine-tune innate immune signaling. Notably, two key gaps in our current understanding warrant further investigation, both of which will require analogous rigorous experimental validation to confirm biological relevance. First, our study focuses on IRF3, but it does not exclude the possibility that gcDDX56 also targets other IRF family members (e.g., IRF7)—a critical question given IRF7’s central role in amplifying type I IFN responses. Second, the molecular cues that drive heightened gcDDX56 activity during GCRV infection remain undefined; resolving this will require dissecting whether activation stems from direct PAMP binding or indirect host stress pathways. These lines of inquiry not only represent tractable future directions but also hold the potential to expand our understanding of how DEAD-box helicases coordinate immune suppression across multiple IRF-dependent signaling axes and how viruses exploit such helicases to subvert host defense.

## MATERIALS AND METHODS

### Cells and virus

*Ctenopharyngodon idellus* kidney (CIK) cells were maintained at 26–28°C with 5% CO_2_ in minimum essential medium (MEM) (Gibco, USA) supplemented with 10% fetal bovine serum (FBS) (Sijiqing, China), 100 U/mL penicillin, and 100 mg/mL streptomycin. Grass carp reovirus (GCRV-873) was amplified in CIK cells using MEM supplemented with 2% FBS and stored at −80°C.

### Plasmid construction and transfection

Based on the sequences from the GenBank database (No. XM_051910428, XM_051872553, XM_051893082, and XM_051894116), the coding regions (CDS) of gcDDX56, gcKPNB1, gcKPNB2, and gcKPNB3 were cloned from cDNA extracted from the liver of grass carp. Constructs gcDDX56-FLAG and gcKPNB3-FLAG were generated using the primers gcDDX56-F1/R1 and gcKPNB3-F1/R1, respectively, and subsequently cloned into the p3 × FLAG-CMV-14 vector (Sigma-Aldrich). Similarly, constructs gcDDX56-HA, gcIRF3-HA, gcKPNB1-HA, gcKPNB2-HA, and gcKPNB3-HA were created using the primers gcDDX56-F2/R2, gcIRF3-F/R, gcKPNB1-F/R, gcKPNB2-F/R, and gcKPNB3-F2/R2 and cloned into pcDNA3.1-HA (Invitrogen). Plasmids RIG-I-FLAG, MDA5-FLAG, MAVS-FLAG, TBK1-FLAG, IRF3-FLAG, and IRF7-FLAG were previously prepared and stored (44–46). The primers are listed in Table S1. Plasmid transfection into CIK cells was performed using NEO (Neofect Biotech, Beijing) following the manufacturer’s protocol.

Truncated and deletion mutants, including gcDDX56-DEAD-FLAG, gcDDX56-Helicase C-FLAG, gcDDX56-C-FLAG, gcDDX56-ΔNLS(340-357)-FLAG, gcDDX56-ΔNLS(526-572)-FLAG, gcKPNB3-KAP95-FLAG, and gcKPNB3-C-FLA, were amplified with primers gcDDX56-DEAD-F/R, gcDDX56-Helicase C-F/R, gcDDX56-C-F/R, gcDDX56-ΔNLS(340-357)-F1/R1/F2/R2, gcDDX56-ΔNLS(526-572)-F/R, gcKPNB3-KAP95-F/R, and gcKPNB3-C-F/R, respectively, and cloned into p3 × FLAG-CMV-14.

### Antibodies and reagents

The anti-FLAG mouse monoclonal antibody (mAb) (#F3165) and the FLAG immunoprecipitation kit were purchased from Sigma-Aldrich. The anti-HA rabbit polyclonal antibody (polyAb) (#51064-2-AP), anti-GAPDH mouse monoclonal antibody (mcAb) (#60004-1-Ig), and anti-Alpha Tubulin recombinant antibody (RecAb) (#80762-1-RR) were obtained from Proteintech. The anti-HDAC1 rabbit polyclonal antibody (PAb) (#ab41407) was acquired from Abcam. Additionally, the anti-NS38, anti-NS80, anti-VP3, and anti-VP5 polyclonal rabbit antibodies against the GCRV-873 strain, as well as the anti-gcIRF3 polyclonal rabbit antibody, were prepared previously and stored in our laboratory (25). The goat anti-mouse immunoglobulin-horseradish peroxidase (Ig-HRP) conjugate secondary antibody, goat anti-rabbit Ig-HRP conjugate secondary antibody, Alexa Fluor 488-conjugated secondary antibody against mouse IgG, Alexa Fluor 594-conjugated secondary antibody against mouse/rabbit IgG, Hoechst 33342, the subcellular protein fractionation kit, protease inhibitor cocktail, TRIzol reagent, and RevertAid First-Strand cDNA Synthesis Kit were purchased from Thermo Fisher Scientific.

### Sequence analysis and phylogenetic analysis

Protein conserved domains were predicted using the Conserved Domain Database (CDD) analysis from NCBI. Multiple alignments of amino acid sequences were conducted using ClustalW and GeneDoc. The phylogenetic tree of DDX56 was constructed using the Neighbor-Joining method in MEGA 11.

### Knockdown of gcDDX56 and gcKPNB3 by siRNA

Transient knockdown of gcDDX56 or gcKPNB3 was performed using siRNAs specifically targeting their mRNAs. Three siRNA sequences, each targeting distinct regions of gcDDX56 or gcKPNB3, were synthesized by Sangon Biotech (Shanghai, China). To evaluate silencing efficiency, CIK cells cultured in 6-well plates were transfected with 100 nM gcDDX56 siRNA, gcKPNB3 siRNA, or control siRNA. At 24 h post-transfection, cells were harvested for qRT-PCR analysis.

### GCRV infection in CIK cells

To assess the role of gcDDX56 overexpression or knockdown in GCRV infection, CIK cells were seeded in 24-well plates overnight, then transfected with 800 ng FLAG empty vector or gcDDX56-FLAG, along with 100 nM siRNA control or si-gcDDX56. After 36 h post-transfection, cells were infected with GCRV at an MOI of 1 or 0.1 at 26–28°C, or left untreated. At 24 hpi, cells were fixed with 4% paraformaldehyde (PFA), stained with 1% crystal violet, and photographed. Supernatants were collected to determine GCRV titers via the TCID_50_ assay: CIK cells seeded in 96-well plates for 24 h were infected with 10-fold serially diluted viral samples (in MEM) for 60 min. After removing inocula, cells were cultured in MEM with 2% FBS. After 3 to 4 days, wells with CPE were counted, and TCID_50_ was calculated using the Reed–Muench formula (47).

To further investigate the effects of gcDDX56 and gcKPNB3 overexpression or knockdown on GCRV replication, CIK cells in 24-well plates were transfected with combinations of constructs: YFP-FLAG plus si-RNA-control, gcDDX56-FLAG plus si-RNA-control, gcKPNB3-FLAG plus si-RNA-control, gcDDX56-FLAG plus gcKPNB3-FLAG, gcDDX56-FLAG plus si-gcKPNB3-1, gcKPNB3-FLAG plus si-gcDDX56-2, YFP-FLAG plus si-gcDDX56-2, YFP-FLAG plus si-gcKPNB3-1, and si-gcDDX56-2 plus si-gcKPNB3-1. Transfections used 400 ng plasmid and 100 nM siRNA per well. At 24 h post-transfection, cells were infected with GCRV (MOI of 1) for 12 h, and supernatants were collected to determine GCRV titers via TCID₅₀ as described above.

### Immunofluorescence assays

To visualize the subcellular localization of gcDDX56, CIK cells seeded in 24-well plates overnight were transfected with 600 ng FLAG empty vector or gcDDX56-FLAG. After 36 h post-transfection, cells were either infected with GCRV at a MOI of 1 or left untreated, then fixed with 4% PFA at 6, 12, and 24 hpi. After fixation, cells were permeabilized with 0.2% Triton X-100 for 15 min, blocked with 4% bovine serum albumin (BSA) for 1 h, incubated overnight at 4°C with anti-FLAG antibody (1:1000), and then for 1 h at room temperature with Alexa Fluor 488-conjugated secondary antibody against mouse IgG (1:500).

For co-localization analysis of gcDDX56 with GCRV proteins (NS38, NS80, VP3, VP5), CIK cells seeded in 24-well plates for 12 h were transfected with gcDDX56-FLAG. After 36 h post-transfection, CIK cells were infected with GCRV at a MOI of 1, and fixed with 4% PFA for 1 h at 12 hpi. Cells were permeabilized and blocked as above, then incubated overnight at 4°C with anti-FLAG, rabbit anti-NS38, anti-NS80, anti-VP3, or anti-VP5 antibodies (1:500), followed by a 1-hour incubation at room temperature with Alexa Fluor 488-conjugated secondary antibody or Alexa Fluor 594-conjugated secondary antibody against rabbit IgG (1:500).

To assess gcDDX56-mediated effects on gcIRF3 localization, CIK cells seeded in 24-well plates overnight were transfected with 600 ng of FLAG or gcDDX56-FLAG, along with 600 ng of gcIRF3-HA. After 36 h post-transfection, cells were infected with GCRV (MOI = 1) or left untreated, then fixed with 4% PFA at 12 hpi. After permeabilization and blocking, cells were incubated overnight at 4°C with anti-FLAG and anti-HA antibodies (1:1000), followed by Alexa Fluor 488- and 594-conjugated secondary antibodies for 1 h at room temperature.

For co-localization analysis of gcDDX56, gcIRF3, and gcKPNB3, CIK cells seeded overnight in 24-well plates were transfected with indicated plasmids. At 36 h post-transfection, cells were infected with GCRV (MOI = 1) or left untreated, fixed with 4% PFA at 12 hpi, and processed as above using anti-FLAG and anti-HA antibodies followed by Alexa Fluor 488- and 594-conjugated secondary antibodies.

To determine the role of putative NLS in gcDDX56 nuclear localization, CIK cells seeded overnight in 24-well plates were transfected with full-length gcDDX56-FLAG or NLS deletion mutants (gcDDX56-ΔNLS(340–357)-FLAG, gcDDX56-ΔNLS(526–572)-FLAG, gcDDX56-ΔNLS(340–357&526–572)-FLAG). At 24 h post-transfection, cells were infected with GCRV (MOI = 1) for 12 h, fixed with 4% PFA for 1 h, permeabilized with 0.2% Triton X-100 for 15 min, and blocked with 5% BSA for 1 h. Cells were incubated overnight at 4°C with anti-FLAG antibody, followed by a 1-hour incubation at room temperature with Alexa Fluor 488-conjugated anti-mouse IgG.

To investigate effects of gcDDX56 and its mutants (DEAD, Helicase C, C-terminal) on gcKPNB3 and gcKPNB3-C localization during GCRV infection, CIK cells seeded overnight in 24-well plates were transfected with the indicated plasmids. At 24 h post-transfection, cells were infected with GCRV (MOI = 1) for 12 h, fixed with 4% PFA for 1 h, and processed as above. Cells were incubated overnight at 4°C with anti-FLAG and anti-HA antibodies, followed by a 1-hour incubation at room temperature with Alexa Fluor 488-conjugated anti-mouse IgG and Alexa Fluor 594-conjugated anti-rabbit IgG.

In all experiments, cells were stained with Hoechst 33342 for 15 min in the dark, followed by three 5-minute washes in PBST after each step. Images were acquired using a Leica SP8 confocal microscope.

### Co-immunoprecipitation assay and western blotting

For Co-IP assays, CIK cells seeded in 10 cm diameter dishes overnight were transfected with indicated plasmids or siRNAs for 36 h, followed by infection with GCRV at a MOI of 1 for 12 h. Cells were lysed overnight in 600 µL of IP lysis buffer containing 1% protease inhibitor cocktail. After centrifugation at 12,000 × *g* for 10 min at 4°C to remove debris, Co-IP was performed using a FLAG-tagged Protein Immunoprecipitation Kit according to the manufacturer’s instructions. Agarose beads were washed three times with ice-cold PBS, incubated with cell lysate overnight, and then washed three times before being centrifuged at 1,000 × *g* for 1 min. Precipitates were resuspended in ice-cold PBS for Western blotting.

To investigate gcDDX56-mediated gcIRF3 degradation, CIK cells seeded in six-well plates were co-transfected with 1.5 µg YFP-FLAG or gcDDX56-FLAG plus 1.5 µg gcIRF3-HA for 36 h, followed by infection with GCRV (MOI = 1) and treatment with 100 mg/mL of cycloheximide (CHX, S7418), an inhibitor of protein synthesis. At 0, 4, 8, and 12 h post-treatment, cells were harvested for protein extraction and Western blot analysis.

To dissect the mechanism of gcDDX56-induced gcIRF3 degradation, CIK cells seeded in six-well plates were transfected with 3 µg of YFP-FLAG or gcDDX56-FLAG for 36 h, followed by infection with GCRV at a MOI of 1 and treatment with 40 µM MG132 (S2619), 10 mM 3-methyladenine (3-MA; S2767), 40 µM chloroquine (CQ; C6628), or 40 mM NH_4_Cl for 6 h. Cells were processed for protein extraction and Western blot analysis.

To map interaction domains between gcDDX56 and gcKPNB3, CIK cells in 10 cm diameter dishes were co-transfected with 6 µg each of YFP-FLAG, gcDDX56-FLAG, gcDDX56-DEAD-FLAG, gcDDX56-Helicase C-FLAG, or gcDDX56-C-FLAG plus gcKPNB3-HA for 36 h. For gcKPNB3 domain mapping, cells were co-transfected with 6 µg each of YFP-FLAG, gcKPNB3-FLAG, gcKPNB3-KAP95-FLAG, or gcKPNB3-C-FLAG plus gcDDX56-HA for 36 h. All cells were infected with GCRV (MOI = 1) for 16 h, lysed in 600 µL IP lysis buffer with protease inhibitors, and processed for Co-IP and Western blotting as described above.

For Western blotting, lysates were subjected to 10% SDS-PAGE and transferred to 0.45 µm PVDF membranes. Membranes were blocked with 5% nonfat milk in Tris-buffered saline-Tween (TBST, 0.1% Tween) for 1 h, then incubated overnight at 4°C with primary antibodies including anti-FLAG (1:5,000), anti-HA (1:5,000), anti-GAPDH (1:5,000), anti-VP3 (1:5,000), anti-VP5 (1:5,000), anti-NS38 (1:5,000), anti-NS80 (1:5,000), anti-IRF3 (1:2,000), anti-HDAC1 (1:5,000), and anti-Tubulin (1:5,000). After washing three times with TBST, the membrane was incubated with goat anti-mouse IgG-HRP conjugate secondary antibody (1:5,000) for 1 h at room temperature. Bands were detected using Pierce ECL Western Blotting Substrate and the ECL Western blot system (LAS-4000 mini), with protein ratios quantified by Image J.

### Luciferase activity assay

To investigate the effects of gcDDX56 on the promoter activities of IFN1 and IFN3, as well as its influence on the promoter activities of interferons mediated by antiviral genes, including RIG-I, MDA5, MAVS, TBK1, IRF3, and IRF7, CIK cells were seeded overnight and subsequently transfected with the indicated plasmids or siRNA. Each transfection included 250 ng of the gcIFN1pro-luc or gcIFN3pro-luc reporter plasmid (48) and 25 ng of a *Renilla* luciferase reporter plasmid. After 36 h post-transfection, the cells were infected with GCRV (MOI = 1) for 12 h. Following infection, the cells were lysed for 20 min using Passive Lysis Buffer, and luciferase activity was measured with the Dual-Luciferase Reporter Assay System (Promega).

### qRT-PCR

To investigate the expression changes of gcDDX56 in response to GCRV infection, CIK cells were seeded in 6-well plates for 24 h and subsequently infected with GCRV or left untreated. RNA was extracted from the cells at 6, 12, and 24 hpi. To assess the impact of the gcIRF3-gcKPNB3 complex on the transcription of genes involved in the RLR antiviral signaling pathway and the downstream interferon-stimulated genes, as well as the role of gcDDX56 in this process, CIK cells were transfected with the specified plasmids or siRNAs after being seeded in 6-well plates. Following 24 h post-transfection, the cells were infected with GCRV at a MOI of 1. RNA extraction was performed using TRIzol reagent, and RNase-free DNase I was employed to eliminate genomic DNA remnants. First-strand cDNAs were synthesized using the RevertAid First-Strand cDNA Synthesis Kit. qRT-PCR was conducted with Fast SYBR Green PCR Master Mix (Bio-Rad) on a CFX96 Touch Real-Time PCR Detection System (Bio-Rad) in 96-well plates. The protocol included preincubation at 95°C for 5 min, followed by 45 cycles of 95°C for 15 s, 56°C for 20 s, and 72°C for 20 s. Each sample was tested in triplicate. The relative mRNA expression was calculated by normalizing the Ct values of target genes against the housekeeping genes β-actin, EF-1α, and GAPDH using the 2^-△△Ct^ method. All primers used for qRT-PCR are listed in Table S1.

### Subcellular fractionation and analysis of gcIRF3 nucleocytoplasmic translocation

To investigate whether gcDDX56 impaired gcIRF3 nuclear translocation, CIK cells seeded in 6-well plates overnight were transfected with 3 µg FLAG/gcDDX56-FLAG or 100 nM of either si-RNA control or si-gcDDX56-2. After 36 h post-transfection, cells were infected with GCRV (MOI = 1) or left untreated, then harvested with trypsin-EDTA at 12 hpi and centrifuged at 500 × for 5 min. Subcellular fractions were separated using a protein fractionation kit: cell pellets were resuspended in cytoplasmic extraction buffer (CEB), incubated at 4°C for 10 min with gentle mixing, and centrifuged at 500 × *g* for 5 min to collect cytoplasmic extract (supernatant). The pellet was resuspended in membrane extraction buffer (MEB), incubated for 10 min, and centrifuged at 3,000 × *g* for 5 min to collect membrane extract (supernatant). Remaining pellets were resuspended in nuclear extraction buffer (NEB), incubated for 30 min, and centrifuged at 5,000 × *g* for 5 min to collect soluble nuclear extract (supernatant). Fractions were analyzed by Western blotting as described above.

To assess effects of gcDDX56 and gcKPNB3 overexpression or knockdown on gcIRF3 protein levels and nucleocytoplasmic translocation during GCRV infection, CIK cells seeded in 6-well plates overnight were co-transfected with 1.5 µg indicated plasmids or 100 nM siRNA. At 36 h post-transfection, cells were infected with GCRV (MOI = 1) or left untreated and then harvested and processed for subcellular fractionation (as described above) at 12 hpi. Fractions were analyzed by Western blotting as described above.

### Statistical analysis

Statistical analysis and graphs were performed and produced using GraphPad Prism 8.0 software. Data from qRT-PCR are presented as mean and SEM. The significance of results was analyzed by an ANOVA or Student’s *t*-test (*P < 0.05; **P < 0.01; ns, not significant).

## Data Availability

All data generated for this study are included in this article and its supplemental material.

## SUPPLEMENTAL MATERIAL

Supplemental material jvi.01654-25-s0001.pdf Figures S1 to S5; Tables S1 and S2.

ASM does not own the copyrights to Supplemental Material that may be linked to, or accessed through, an article. The authors have granted ASM a non-exclusive, world-wide license to publish the Supplemental Material files. Please contact the corresponding author directly for reuse.
