# Supplementary material for: DEAD-box helicase gcDDX56 disrupts IRF3 nuclear import complex and promotes nuclear IRF3 degradation for enhancing GCRV replication
Source: J Virol. 2025 Nov 11;99(12):e01654-25. doi: 10.1128/jvi.01654-25 (PMC12724329; doi:10.1128/jvi.01654-25)
Supplement: Supplemental material — Figures S1 to S5; Tables S1 and S2. [file jvi.01654-25-s0001.pdf]

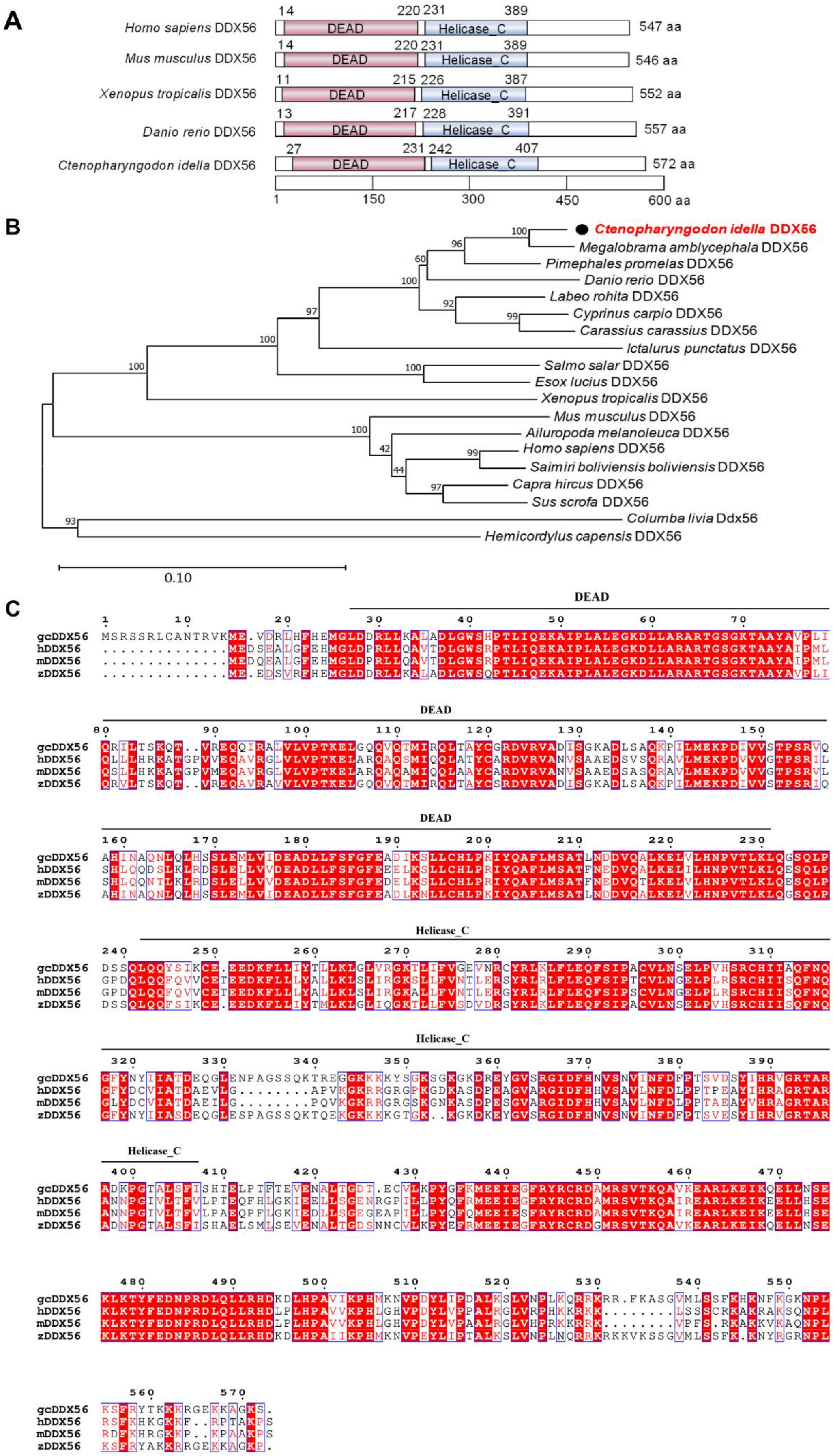
A
Homo sapiens DDX56
14 DEAD 220 231 Helicase_C 389
547 aa
Mus musculus DDX56
14 DEAD 220 231 Helicase_C 389
546 aa
Xenopus tropicalis DDX56
11 DEAD 215 226 Helicase_C 387
552 aa
Danio rerio DDX56
13 DEAD 217 228 Helicase_C 391
557 aa
Ctenopharyngodon idella DDX56
27 DEAD 231 242 Helicase_C 407
572 aa
1 150 300 450 600 aa
B
Ctenopharyngodon idella DDX56
Megalobrama amblycephala DDX56
Pimephales promelas DDX56
Danio rerio DDX56
Labeo rohita DDX56
Cyprinus carpio DDX56
Carassius carassius DDX56
Ictalurus punctatus DDX56
Salmo salar DDX56
Esox lucius DDX56
Xenopus tropicalis DDX56
Mus musculus DDX56
Ailuropoda melanoleuca DDX56
Homo sapiens DDX56
Saimiri boliviensis boliviensis DDX56
Capra hircus DDX56
Sus scrofa DDX56
Columba livia Ddx56
Hemicordylus capensis DDX56
0.10
C
DEAD
Helicase_C
gcDDX56
hDDX56
mDDX56
zDDX56

Supplemental Fig. 1. Structural domain, phylogenetic, and sequence alignment analyses of vertebrate DDX56. (**A**) Domain analysis of vertebrate DDX56. (**B**) Phylogenetic analysis of vertebrate DDX56; tree constructed using the Neighbor-Joining method in MEGA 11. (**C**) Amino acid sequence alignment of gcDDX56 with DDX56 from zebrafish, mouse, and human. DEAD and Helicase C domains are indicated by black lines.

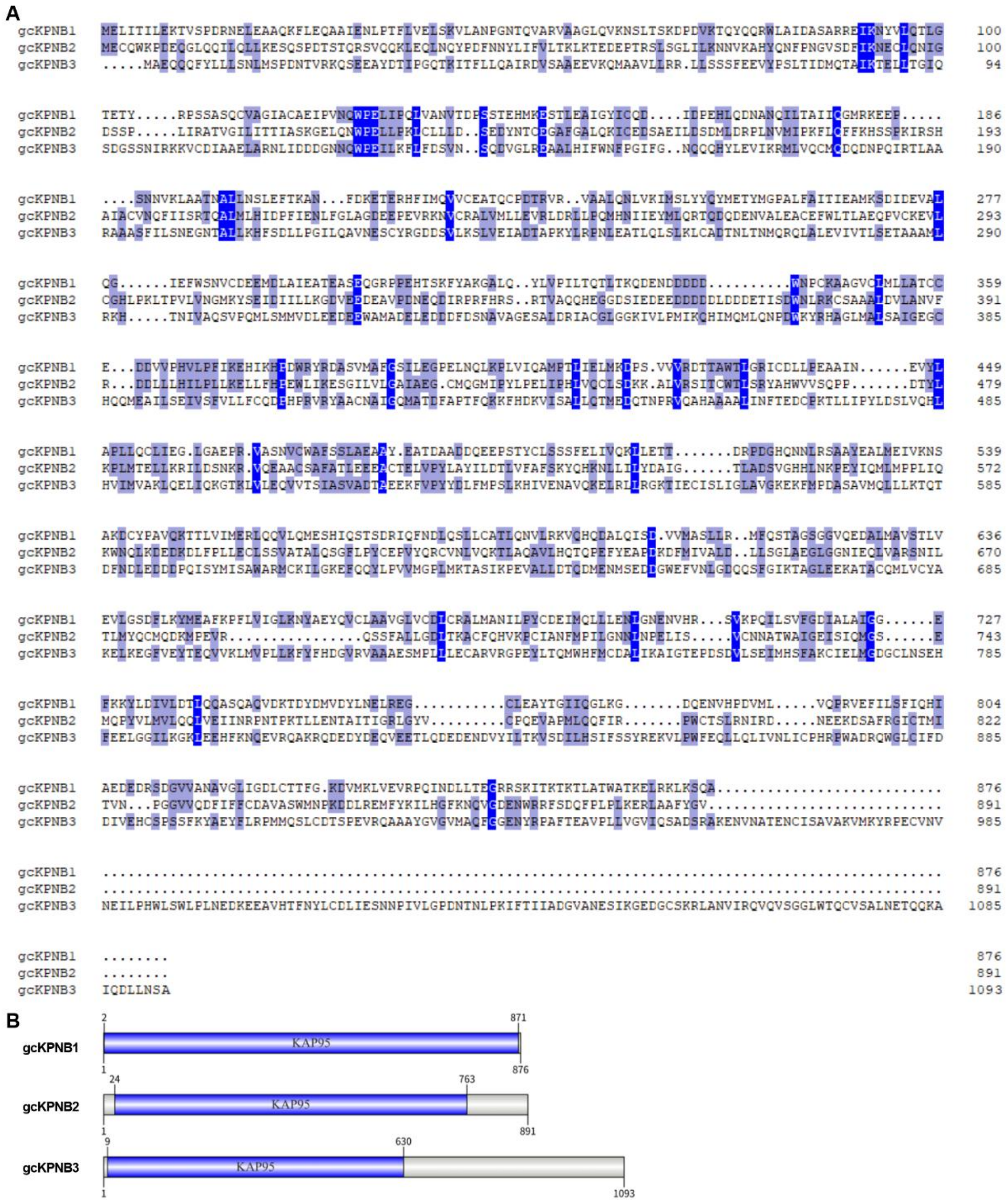

Supplemental Fig. 2. Amino acid sequence alignment and conserved domain analysis of gcKPNB family members. (**A**) Amino acid sequence alignment of gcKPNB1, gcKPNB2, and gcKPNB3. (**B**) Schematic of conserved domain architecture among gcKPNB family members; KAP95 domains are marked in blue.

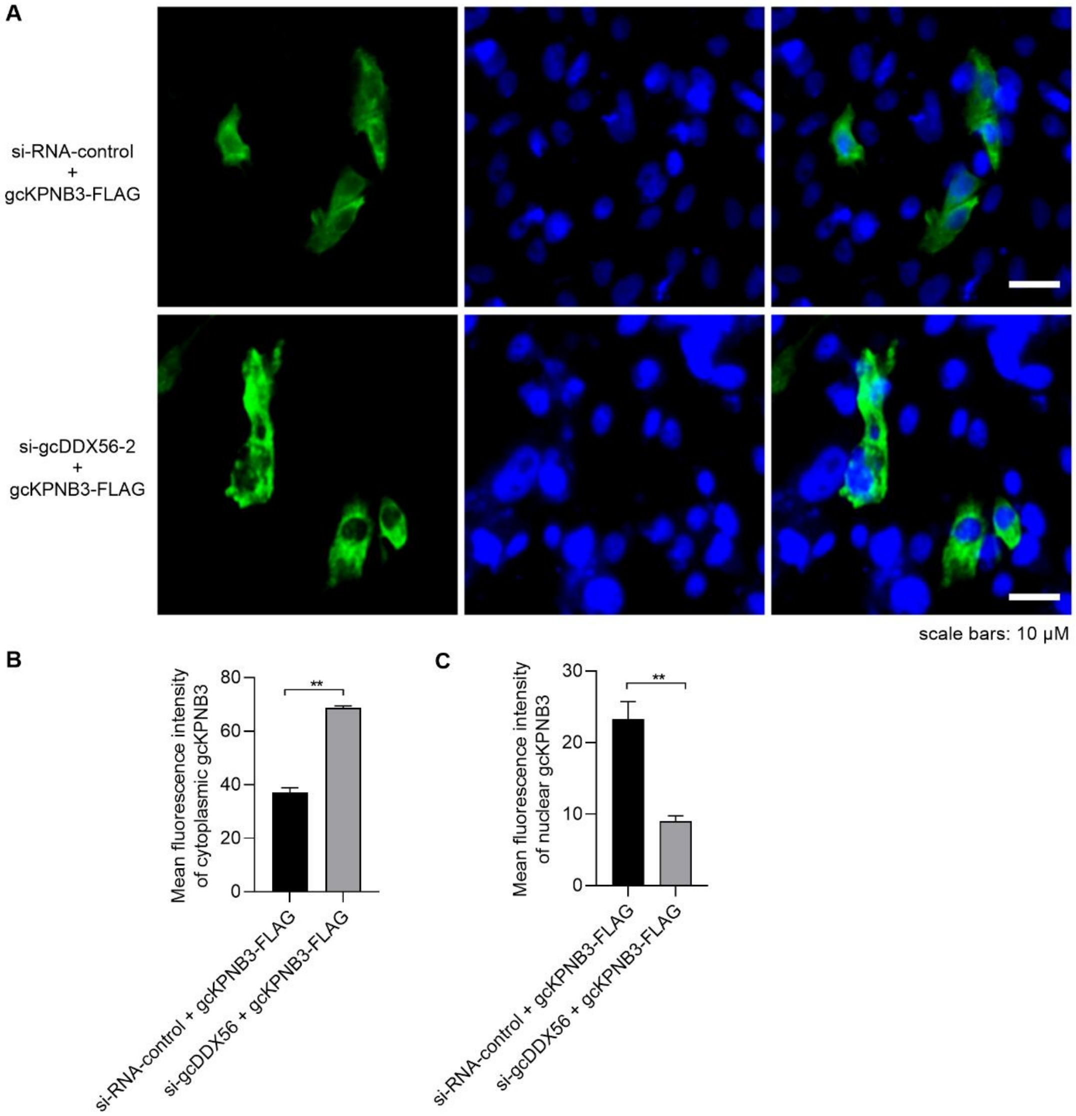

Supplemental Fig. 3. Immunofluorescence analysis of gcKPNB3 nucleocytoplasmic distribution following gcDDX56 knockdown. (**A**) Effect of gcDDX56 knockdown on the nucleocytoplasmic distribution of gcKPNB3. (**B**) gcDDX56 knockdown increases the immunofluorescence intensity of cytoplasmic gcKPNB3. (**C**) gcDDX56 knockdown decreases the immunofluorescence intensity of nuclear gcKPNB3.

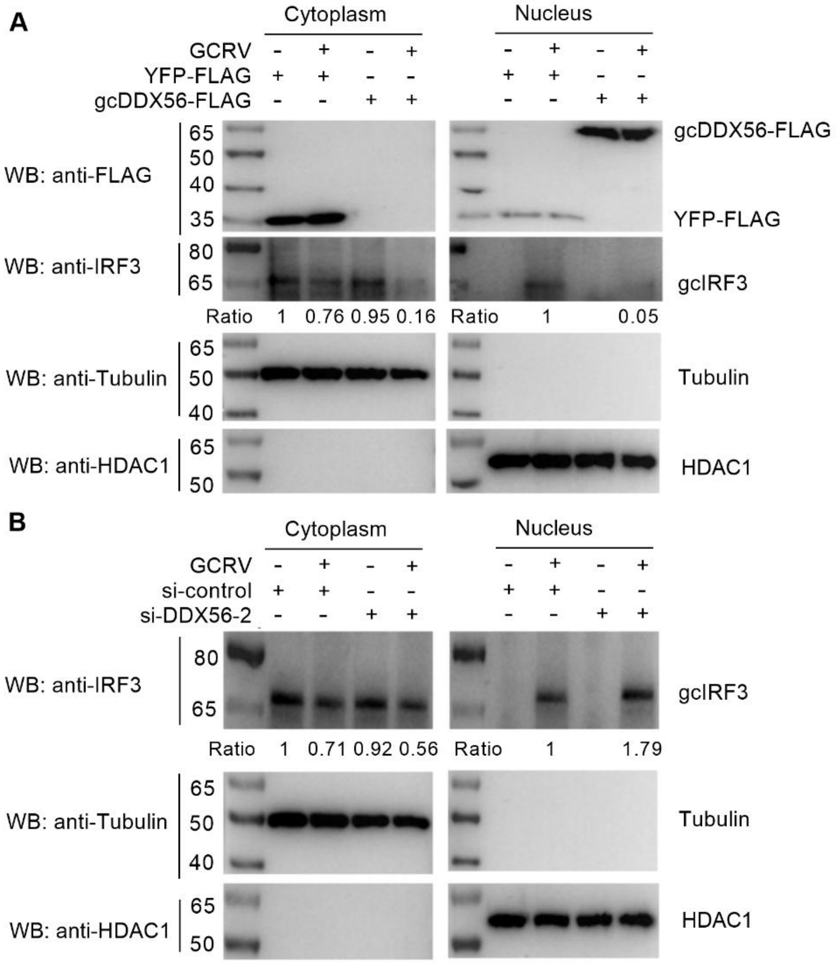

Supplemental Fig. 4. The effect of gcDDX56 on the protein levels of nuclear and cytoplasmic IRF3 during GCRV infection. (**A**) Overexpression of gcDDX56 reduced nuclear and cytoplasmic IRF3 protein levels during GCRV infection. (**B**) Knockdown of gcDDX56 increased nuclear IRF3 protein levels during GCRV infection. For A and B, protein bands were quantified by Image J.

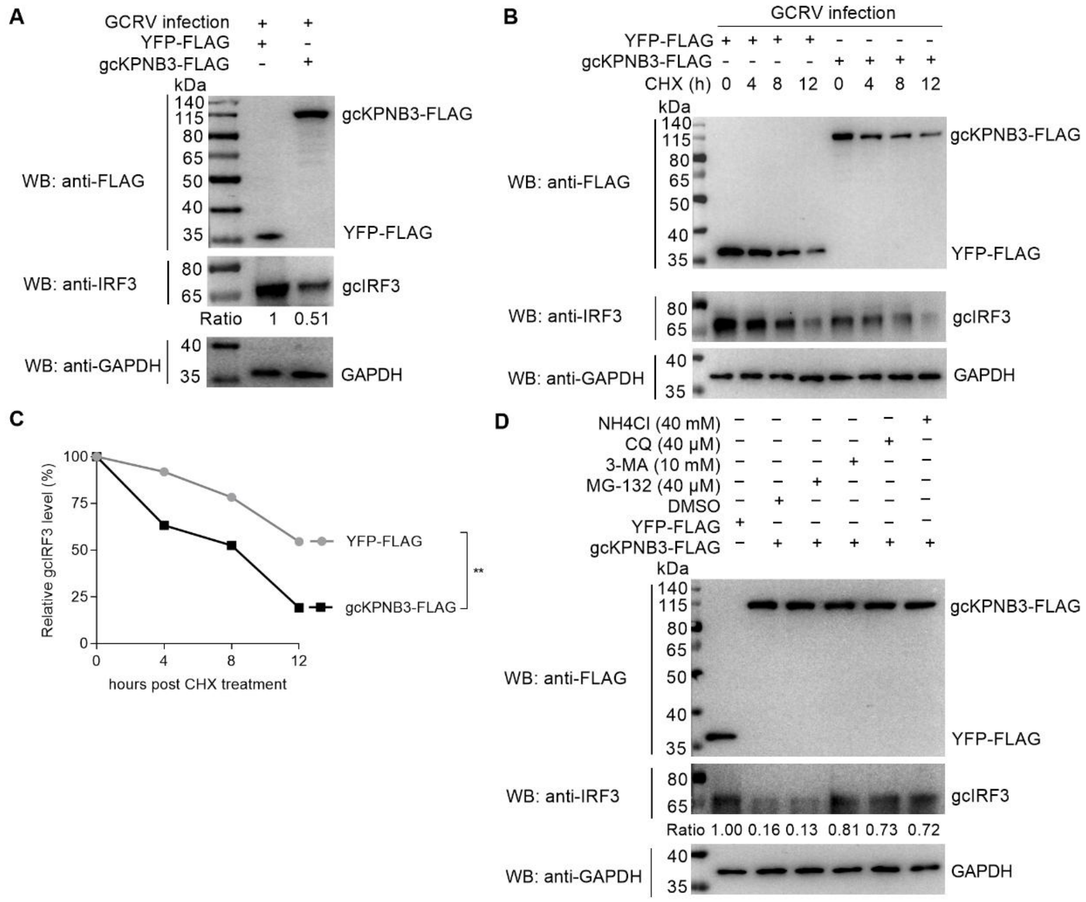

Supplemental Fig. 5. gcKPNB3 mediates gcIRF3 degradation via the autophagy-lysosome pathway during GCRV infection. (**A**) Effect of gcKPNB3 overexpression on endogenous gcIRF3 protein levels in GCRV-infected cells. (**B**) Effect of gcKPNB3 on endogenous gcIRF3 protein turnover following cycloheximide (CHX)-mediated protein synthesis inhibition. (**C**) Relative gcIRF3 protein levels normalized to the housekeeping protein GAPDH. (**D**) gcKPNB3 promotes gcIRF3 degradation via the autophagy-lysosome pathway.

Table S1. The primer sequences used for the present study.

Name	Sequence	Application
gcDDX56-F1	CCAAGCTTATGTCTCGCTCGTCTCGTC	Ligated to p3xFLAG vector
gcDDX56-R1	CGGGATCCGGATTTCCCAGCCTT	
gcKPNB3-F1	CCCAAGCTTATGGCGGAGCAGCAG	
gcKPNB-R1	GCTCTAGAGGCCGAGTTCAGGAGATCC	
gcDDX56-ΔNLS(340-357)-F1	CCAAGCTTATGTCTCGCTCGTCTCGTC	
gcDDX56-ΔNLS(340-357)-F2	GCTGGAAGTTCCCAGGAATATGGCG	
gcDDX56-ΔNLS(340-357)-R1	GGAAACGCCATATTCCTGGGAAC	
gcDDX56-ΔNLS(340-357)-R2	CGGGATCCGGATTTCCCAGCCTTCTTTTCAC	
gcDDX56-ΔNLS(526-572)-F	CCAAGCTTATGTCTCGCTCGTCTCGTC	
gcDDX56-ΔNLS(526-572)-R	CGGGATCCAGGATTGACGAGACTTTTCAGC	
gcDDX56-DEAD-F	CCCAAGCTTATGTCTCGCTCGTCTCGT	
gcDDX56-DEAD-R	CGGGATCCGAGCTTCAGCGTCACCG	
gcDDX56-Helicase C-F	CCCAAGCTTATGGATAAGTTCCTGCTCATCTAC	
gcDDX56-Helicase C-R	CGGGATCCCGCACGAGCCGTTC	
gcDDX56-C-F	CCCAAGCTTATGGATAAACCCGGCACTG	
gcDDX56-C-R	CGGGATCCGGATTTCCCAGCCTTCTTTTC	
gcKPNB3-KAP95-F	CCCAAGCTTATGCTCTTACTGAGCAACCTG	
gcKPNB3-KAP95-R	GCTCTAGATGCAGTCTTCATCAGCGGAC	
gcKPNB3-C-F	CCCAAGCTTATGGCGGAGCAGCAGCAGTTCTACTCCATAAAACCAG	
gcKPNB3-C-R	GCTCTAGAGGCCGAGTTCAGGAGATCC	
gcDDX56-F2	CGGGATCCATGTCTCGCTCGTCTCGTC	Ligated to pcDNA3.1-HA vector
gcDDX56-R2	GCTCTAGAGGATTTCCCAGCCTTCTTTTCAC	
gcIRF3-F	CGGAATTCATGACCCATCCAAAACCGC	
gcIRF3-R	GCTCTAGACTTGGTGTCACACAACTCC	
gcKPNB1-F	GGGGTACCATGGAGCTGATCACAATCCTC	
gcKPNB1-R	GCTCTAGAAGCTTGGCTCTTCAGCTTG	
gcKPNB2-F	GGGGTACCATGGAGTGCCAGTGGAAAC	
gcKPNB2-R	GCTCTAGACACCCCATAGAAGGCTGC	
gcKPNB3-F2	CGGAATTCATGGCGGAGCAGCAG	
gcKPNB3-R2	CCCTCGAGGGCCGAGTTCAGGAGATCC	
si-gcDDX56-1	GUGUCACUUACCCAAGAUC	Synthesizing
si-gcDDX56-2	GUGUCUAAUGUGAUCAACU	

si-gcDDX56-3	GAGGAAAUCGAGGGAUUCA	si-gcDDX56
si-gcKPNB3-1	GCAGCAGUUCUACCUCUUA	Synthesizing si-gcKPNB3
si-gcKPNB3-2	GGACGUCGGUCUCAGAGAA	
si-gcKPNB3-3	GGUCAGACGAAGAUCACAU	
q-*ddx56*-F	GTGAGGAGGAGGATAAGTTC	qRT-PCR
q-*ddx56*-R	ATTCAGAGTTCAGGACACAA	
q-*kpnb3*-F	GAAGCCACTCTCCAACTG	
q-*kpnb3*-R	CACCATCATACTCAACATCTG	
q-*rig-i*-F	ACTACACTGAACACCTGCGGAA	
q-*rig-i*-R	GCATCTTTAGTGCGGGCG	
q-*mda5*-F	CAGGAGCGACTCTTGGACTATG	
q-*mda5*-R	AAAGACGGTTTATTTGAATGGAAG	
q-*mavs*-F	GACCGTAAGAAGTCAGCCTCC	
q-*mavs*-R	CCTGAATAACTCTTGATAGCCCTC	
q-*tbk1*-F	CCAGGAGAAATGTTGGGGC	
q-*tbk1*-R	TGTAGATGTGGTGGAGTGTCGC	
q-*irf3*-F	ACTTCAGCAGTTTAGCATTCCC	
q-*irf3*-R	GCAGCATCGTTCTTGTTGTCA	
q-*irf7*-F	CGCCTGTGTTCGTCACTCGT	
q-*irf7*-R	GGTGGTTGGAAAGCGTATTGG	
q-*ifn1*-F	AAGCAACGAGTCTTTGAGCCT	
q-*ifn1*-R	GCGTCCTGGAAATGACACCT	
q-*ifn3*-F	TACATTTATAGAGACTGCGGGTGG	
q-*ifn3*-R	TGGAGTGTCTGGTAAACAGCCTT	
q-*mx1*-F	CTGGGGAGGAAGTAAAGTGTTCT	
q-*mx1*-R	CAGCATGGATTCTGCCTGG	
q-*mx2*-F	ACATTGACATCGCCACCACT	
q-*mx2*-R	TTCTGACCACCGTCTCCTCC	
q-EF1α-F	CAGCACAAACATGGGCTGGTTC	
q-EF1α-R	ACGGGTACAGTTCCAATACCTCCA	
q-β-actin-F	GGCTGTGCTGTCCCTGTA	
q-β-actin-R	GGGCATAACCCTCGTAGAT	
q-GAPDH-F	AACCTGCCAAGTATGATGAT	
q-GAPDH-R	AAGATAGAGGAGCGACAATC	

Table S2. The effects of gcDDX56 and gcKPNB3 overexpression or knockdown on IRF3 expression and GCRV replication.

	Nuclear IRF3 protein	cytoplasmic IRF3 protein	Total IRF3 protein	GCRV replication
gcDDX56 overexpression alone	down	down	down	Promoting GCRV replication
gcKPNB3 overexpression alone	no effect	down	down	Promoting GCRV replication
Co-expression of gcDDX56 and gcKPNB3	down	down	down	Promoting GCRV replication
gcDDX56 overexpression and gcKPNB3 knockdown	down	up	up	Inhibiting GCRV replication
gcDDX56 knockdown and gcKPNB3 overexpression	up	down	up	Inhibiting GCRV replication
gcDDX56 knockdown alone	up	down	up	Inhibiting GCRV replication
gcKPNB3 knockdown alone	down	up	up	Inhibiting GCRV replication
Co-knockdown of gcDDX56 and gcKPNB3	down	up	up	Inhibiting GCRV replication
